# Mutations in *GFAP* Alter Early Lineage Commitment of Organoids

**DOI:** 10.1002/glia.70049

**Published:** 2025-07-30

**Authors:** Werner Dykstra, Zuzana Matusova, Rachel A. Battaglia, Pavel Abaffy, Nuria Goya‐Iglesias, Dolores Pérez‐Sala, Henrik Ahlenius, Mikael Kubista, R. Jeroen Pasterkamp, Li Li, Jianfei Chao, Yanhong Shi, Lukas Valihrach, Milos Pekny, Elly M. Hol

**Affiliations:** ^1^ Department of Translational Neuroscience, UMC Utrecht Brain Center, University Medical Center Utrecht Utrecht University Utrecht the Netherlands; ^2^ Laboratory of Glial Biology and Omics Technologies Institute of Biotechnology of the Czech Academy of Sciences Vestec Czech Republic; ^3^ Faculty of Science Charles University Prague Czech Republic; ^4^ Department of Cell Biology and Physiology University of North Carolina Chapel Hill North Carolina USA; ^5^ Centro de Investigaciones Biológicas Margarita Salas, C.S.I.C. Madrid Spain; ^6^ Stem Cells, Aging and Neurodegeneration, Lund Stem Cell Center, Department of Experimental Medical Science, Faculty of Medicine Lund University Lund Sweden; ^7^ Laboratory of Gene Expression Institute of Biotechnology of the Czech Academy of Sciences Vestec Czech Republic; ^8^ Department of Neurodegenerative Diseases Beckman Research Institute of City of Hope Duarte California USA; ^9^ Department of Cellular Neurophysiology Institute of Experimental Medicine CAS Prague Czech Republic; ^10^ Laboratory of Astrocyte Biology and CNS Regeneration, Center of Brain Repair, Department of Clinical Neuroscience Institute of Neuroscience and Physiology, Sahlgrenska Academy at the University of Gothenburg Gotenburg Sweden

**Keywords:** Alexander disease, GFAP, iPSCs, lineage commitment, neural organoids

## Abstract

Glial fibrillary acidic protein (GFAP) is a type‐3 intermediate filament protein mainly expressed in astrocytes in the central nervous system. Mutations in *GFAP* cause Alexander disease (AxD), a rare and fatal neurological disorder. How exactly mutant GFAP eventually leads to white and gray matter deterioration in AxD remains unknown. GFAP is known to be expressed also in neural precursor cells in the developing brain. Here, we used AxD patient‐derived induced pluripotent stem cells (iPSCs) to explore the impact of mutant GFAP during neurodifferentiation. Our results show that GFAP is already expressed in iPSCs. Moreover, we have found that mutations in GFAP can severely affect neural organoid development through altering lineage commitment in embryoid bodies. Together, these results support the notion that GFAP plays a role as an early modulator of neurodevelopment.

## Main Points

- Mutations in *GFAP* alter lineage commitment of organoids.
- The severity of this commitment defect is dependent on method of embryoid body formation.
- GFAP is expressed in iPSCs and during early stages of organoid development.

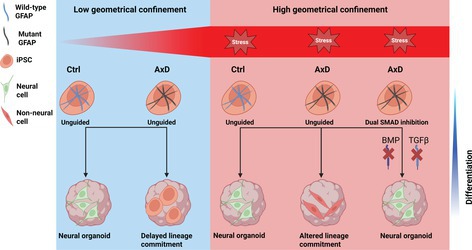

## 1 Introduction

Intermediate filaments (IFs) are components of the cytoskeleton and, as such, regulate cellular dynamics (Herrmann and Aebi 2000, 2004, 2016). Many types of IFs exist, and the composition of different IFs determines a cell's molecular and structural capabilities (Coulombe and Wong 2004). During development, as stem cells generate progeny, differentiated cells start to express IFs that parallel their cellular state (Emerson 1988; Erickson et al. 1987). This suggests that IF function is intertwined with specific cellular functions as well as cell identity during development. Indeed, it has recently been shown that keratins are cell fate determinants in the mammalian embryo (Lim et al. 2020). In the developing central nervous system (CNS), radial glial cells (RGCs) give rise to all neurons and macroglia (Hansen et al. 2010; Kriegstein and Alvarez‐Buylla 2009). They populate the ventricular zone (VZ) and extend processes towards the cortical plate. These processes require flexibility as well as strength, which is provided in part by cytoplasmic intermediate filaments (Arellano et al. 2021). For example, RGCs highly express vimentin and nestin, as well as lower levels of glial fibrillary acidic protein (GFAP) and synemin that together form filaments to provide structure, motility, and function. As RGCs differentiate into astrocytes after neurogenesis has been largely completed, vimentin and nestin are gradually replaced by GFAP, making this type‐3 intermediate filament protein a canonical marker for astrocytes (Hol and Pekny 2015; Middeldorp and Hol 2011; Moeton et al. 2016).

Besides their role during development, many IFs are involved in disease processes (Omary et al. 2004). For instance, mutations in keratins can cause skin diseases (Coulombe et al. 1991) and certain lamin A mutations cause progeria (Eriksson et al. 2003). In the CNS, however, contributions of tissue‐specific IFs to neurodevelopmental disorders have not been described so far. This could be either due to a potential very early, lethal effect of mutations in these IFs or due to a lack of a clearly identifiable effect. However, the effect of such mutations cannot be underestimated. For instance, single point mutations in GFAP can cause Alexander disease (AxD), a rare and fatal disorder of astrocytes that primarily affects children (Brenner et al. 2001). The effect of *GFAP* mutations is likely a gain‐of‐function, because apart from increased hippocampal neurogenesis (Larsson et al. 2004; Wilhelmsson et al. 2012), more pronounced memory extinction (Wilhelmsson et al. 2019) and hypersensitivity to traumatic cerebrospinal injury (Nawashiro et al. 1998), *GFAP* ^−/−^ mice do not exhibit any major phenotypes (Gomi et al. 1995; Pekny et al. 1995), even when vimentin, which partially compensates for the absence of GFAP, is also genetically removed (Ridge et al. 2022). Interestingly, the absence of GFAP on the vimentin null background alters responses to several challenges to the CNS, such as neurotrauma (Cho et al. 2005; Grosche et al. 2013; Lundkvist et al. 2004; Wilhelmsson et al. 2004), CNS ischemia (Aswendt et al. 2022; Järlestedt et al. 2010; Li et al. 2008; Verardo et al. 2008) or neurodegenerative diseases (Kamphuis et al. 2015; Kraft et al. 2013; Macauley et al. 2011). However, rodents carrying AxD mutations in *GFAP* faithfully recapitulate several aspects of AxD that include astrocyte pathology, neurodegeneration, and death (Hagemann et al. 2006, 2009, 2013, 2021; Jany et al. 2013; Pajares et al. 2023; Pekny et al. 2016; Wang et al. 2022). This suggests that whereas a lack of GFAP does not result in any gross abnormalities, with the exception of adult hippocampal neurogenesis (Hol and Pekny 2015; Larsson et al. 2004; Lebkuechner et al. 2015; Pekny and Pekna 2014; Pekny et al. 2019; Widestrand et al. 2007; Wilhelmsson et al. 2012), single point mutations in *GFAP* are sufficient to induce severe neurological abnormalities and dysfunctions. Since AxD is classified as an astrocytopathy (Borrett and Becker 1985; Brenner et al. 2009), in which mutant GFAP‐expressing astrocytes are drivers of white and gray matter deterioration (Messing et al. 2012), most research using animal models has focused on astrocytes. However, GFAP is also expressed in the mammalian brain, already during early developmental stages, by RGCs (Choi 1988; Middeldorp et al. 2010) and multipotent neural stem/progenitor cells (Imura et al. 2003). Therefore, mutant GFAP could potentially disrupt neurodevelopment. Interestingly, previous research showed no obvious neurodevelopmental disruptions in a mouse model of AxD (Hagemann et al. 2006). However, it has recently been shown that the onset of GFAP expression in humans starts at the onset of corticogenesis, whereas in rodents it starts when corticogenesis has been completed (Arellano et al. 2021). Such a species‐specific difference in onset of GFAP expression renders human cells and specifically human neural organoids particularly useful to study any potential human‐specific AxD RGC pathology. In support of this, we have shown that the AxD‐causing R239C mutation in *GFAP* caused aberrant neurodevelopment in neural organoids at a developmental stage when astrocytes are typically present, as well as in co‐cultures of neurons and R239C mutation‐carrying astrocytes derived from AxD‐patient‐derived iPSCs (Matusova et al. 2025).

To investigate whether other AxD mutations in *GFAP* also resulted in abnormal organoid development and at what stage this altered development is initiated, we generated neural organoids from iPSCs derived from three different AxD patients, carrying different heterozygous mutations in *GFAP*. Remarkably, we found that GFAP is already expressed by iPSCs and that mutant *GFAP* disrupts early lineage commitment of embryoid bodies. Changing the properties of embryoid body formation to a less geometrically confining approach or forcing GFAP mutant embryoid bodies to commit to the neuroectoderm through dual SMAD inhibition, prevented altered lineage commitment but produced neural organoids that were delayed in acquiring neuroectodermal fate. These results point to a novel function of GFAP and show that single point mutations in *GFAP* can have large effects on organoid development, especially under geometrically confining conditions.

## 2 Methods

### 2.1 iPSCs

R239C‐AxD‐1 and R239R‐Ctrl iPSCs were obtained from the University of North Carolina (USA) (Battaglia et al. 2019), R239C‐AxD‐2 iPSCs were obtained from the Beckman Research Institute (USA) (Li et al. 2018) and R88C‐AxD and R416W‐AxD iPSCs were obtained from WiCell (USA) (Jones et al. 2018). Healthy control iPSCs were previously described (Ormel et al. 2018). Genomic characterization, characterization of pluripotency and the undifferentiated state, confirmation of cell type, and molecular characterization were previously performed (Battaglia et al. 2019; Jones et al. 2018; Li et al. 2018; Ormel et al. 2018). iPSCs were maintained in Stemflex medium (Life technologies, A3349401) on Geltrex‐coated (Life technologies, A1413202) dishes in feeder‐free conditions at 37°C with 5% CO_2_. Medium was changed daily and cells were split in a 1:6 ratio in Stemflex medium containing 5 μM Y27632 (Axon Biochemicals, AXON 1683) as soon as they reached 80% confluency by incubating them with 0.5 mM EDTA in for 3 min at 37°C. After 24 h, medium was changed to regular Stemflex medium. Cells were frozen at −80°C in freezing medium containing 90% FBS (ThermoFisher Scientific, 10,082,147) and 10% DMSO. For long term storage, cells were transferred to liquid nitrogen tanks. For thawing, cells were taken from the liquid nitrogen tank and thawed at 37°C. Cells were then spun down at 200×G for 3 min and gently resuspended in StemFlex medium containing 5 μM Y27632. The next day, medium was changed to regular StemFlex medium. The number of passages were kept below 40. Routine testing for mycoplasma (Lonza, LT07‐318, Lonza Bioscience Solutions, Basel, Switzerland) was performed and cells were visually inspected to check for bacterial infections.

### 2.2 Generation of Unguided Neural Organoids

Unguided neural organoids were generated as previously described (Lancaster et al. 2013; Ormel et al. 2018). Briefly, at day 0, iPSCs that reached 80% confluency were dissociated into single cells following a 2‐min incubation period with 0.5 mM EDTA in PBS and a 4‐min incubation period with Accutase at 37°C. For Aggrewell800 organoids, 3.5 × 10^6^ (regular) or 1.35 × 10^6^ iPSCs were seeded in a well of an Aggrewell800 plate (STEMCELL technologies, 34,811) in 3 mL human embryonic stem (HES) medium consisting of DMEM/F‐12 (ThermoFisher Scientific, 31,330,038), 20% KOSR (Life Technologies,10,828,028), 3% FBS (ThermoFisher Scientific, 10,082,147), 1% Glutamax (ThermoFisher Scientific, 35,050,061), 1% NEAAs (ThermoFisher Scientific, 11,140,035), 0.1 mM 2‐Mercaptoethanol (Merck, 8,057,400,005) supplemented with 4 ng/mL bFGF and 50 μM Y27632 (Axon Biochemicals, AXON 1683) (HES4+). Embryoid bodies were allowed to form at 37°C with 5% CO_2_. After 24 h, 1.5 mL of medium was replaced with fresh HES4+ medium. At day 2, properly formed embryoid bodies were transferred to an ultra‐low attachment round‐bottom shaped 96‐well plate (Corning, 3474) containing a total of 150 μL of HES4+ medium. For direct seeding organoids, ~11.5 K iPSCs were allowed to form an embryoid body at 37°C with 5% CO_2_ in a well of an ultra‐low attachment round‐bottom shaped 96‐well plate (Corning, 3474) containing a total of 150 μL HES4+ medium. At day 2, 100 μL of medium was replaced by 150 μL of fresh HES4+ medium. For Aggrewell800 versus direct seeding method experiments, organoids for both protocols were generated from the same iPSC suspension. From day 4 onwards, the Aggrewell800 protocol and the direct seeding protocol were the same. At day 4, 150 μL of medium was removed and replaced by 150 μL HES medium. At day 6, 150μL of medium was replaced with 150 μL neural induction medium (NIM) consisting of DMEM/F‐12 (Thermo Fisher Scientific, 31,330,038), 1% N2 (Life Technologies, 17,502,048), 1% l‐glutamine (Life Technologies, 25,030,024), 1% NEAA (Thermo Fisher Scientific, 11,140,035) and 0.5 μg/mL heparin (Sigma‐Aldrich, H3149). At day 8, 150 μL of medium was replaced with fresh NIM. At day 9, organoids were harvested for analysis. At least 4 independent batches were used for experiments.

### 2.3 Generation of Cortical Organoids

Cortical organoids were generated as described previously, with minor adaptations (Gordon et al. 2021). Briefly, iPSCs that reached 80% confluency were dissociated into single cells following a 7‐min incubation period with Accutase at 37°C. After counting, 3.5 × 10^6^ iPSCs were seeded in a well of an Aggrewell800 plate (STEMCELL technologies, 34,811) in 2 mL HES4+ medium. Embryoid bodies were allowed to form at 37°C with 5% CO_2_. After 24 h, 1.5 mL of medium was replaced with fresh HES4+ medium. At day 2, properly formed embryoid bodies were transferred to an ultra‐low attachment round‐bottom shaped 96‐well plate (Corning, 3474) containing a total of 150 μL of HES medium supplemented with two SMAD pathway inhibitors—Dorsomorphin (2.5 μM; Tocris 3093) and SB43125 (10 μM; Axon biochemicals 1661) to start neural induction. At day 4, 100 μL of medium was replaced with 150 μL of fresh medium. At days 6 and 8, 150 μL of medium was replaced by 150 μL of Neural media consisting of Neurobasal media (ThermoFisher Scientific, 21,103,049), 2% B27‐vitamin A (ThermoFisher Scientific, 12,587,001), 1% Penicillin–Streptomycin (Life Technologies, 15,140,122) and 1% l‐glutamine (Life Technologies, 25,030,024) supplemented with 20 ng/mL EGF (R&D systems 236‐EG) and 20 ng/mL bFGF (Peptro‐Tech 100‐18B). Cortical organoids were harvested at day 9. At least 4 independent batches were used for experiments.

### 2.4 Dual SMAD Inhibition Experiments

At day 0, iPSCs that reached 80% confluency were dissociated into single cells following a 2‐min incubation period with 0.5 mM EDTA in PBS and a 4‐min incubation period with Accutase at 37°C. 3.5 × 10^6^ iPSCs were seeded in a well of an Aggrewell800 plate (STEMCELL technologies, 34,811) in 3 mL human embryonic stem HES4+ medium supplemented with 50 μM Y27632 (Axon Biochemicals, AXON 1683) and two SMAD pathway inhibitors—Dorsomorphin (2.5 μM; Tocris 3093) and SB43125 (10 μM; Axon biochemicals 1661) (HES4+—DSi medium). At day 2, embryoid bodies were transferred to an ultra‐low attachment round‐bottom shaped 96‐well plate (Corning, 3474) containing a total of 150 μL of HES4+—DSi medium. From day 4 onwards, the protocol was similar to that of unguided neural organoids. At least 4 independent batches were used for experiments.

### 2.5 Generation of R239C‐GFAP‐KO iPSC Line Using CRISPR/Cas9

The GFAP KO iPSC line was generated from the AxR‐R239C‐1 iPSC line using a Cas9‐expressing vector, in which a gRNA targeting exon 1 of *GFAP* was cloned. The gRNA was designed with Off‐Spotter (https://cm.jefferson.edu/Off‐Spotter/) and selected according to MIT and CFD specificity score (Doench et al. 2016). gRNAs were cloned into pSpCas9(BB)‐2A‐GFP (Addgene, #48138) after Bpil (Thermo Fisher Scientific, ER1011) digestion and, using a Maxiprep kit (LabNed, LN2400008), plasmids were isolated to verify the sequence (5′‐CTCCCGACCCGGGTGGATTT‐3′) with Sanger sequencing (Macrogen, Amsterdam, The Netherlands). The transfection was performed using a modified protocol of (Yumlu et al. 2017). Three days before transfection, 100,000 single AxD‐R239C‐1 iPSCs were plated per well of a Geltrex‐coated (Life technologies, A1413202) 6‐well plate (Co rning, 3516) in Stemflex medium (Life technologies, A3349401) containing 5 μM Y‐27632 ROCK‐inhibitor (Axon Biochemicals, AXON 1683). On day 0, 2 μg of the plasmid, 2.5 μL PLUS reagent, and 5 μL Lipofectamine LTX reagent (Invitrogen, 15,338,100) were mixed with 250 μL Opti‐MEM (Gibco, 31,985,062) and incubated at room temperature for 10 min, before being diluted in 2 mL Opti‐MEM (Gibco, 31,985,062) with 5 μM Y‐27632 ROCK‐inhibitor (Axon Biochemicals, AXON 1683). This mixture was added to the well, after aspirating the medium, and then incubated for 3 h at 37°C. Stemflex medium (Life technologies, A3349401) with 5 μM Y‐27632 ROCK‐inhibitor (Axon Biochemicals, AXON 1683) was added and incubated overnight at 37°C. The next day, transfection reagents were removed and fresh Stemflex medium (Life technologies, A3349401) was added. On day 2, fluorescence associated cell sorting (FACS) was performed by dissociating the transfected cells with Accutase (tebu‐bio, AT‐104) as described before, collecting them in Stemflex (Life technologies, A3349401), and spinning them down for 4 min at 200×g. Cell pellets were resuspended in FACS medium consisting of Stemflex (Life technologies, A3349401) supplemented with 1% Penicillin–Streptomycin (Life Technologies, 15,140,122) and 5 μM Y‐27632 ROCK‐inhibitor (Axon Biochemicals, AXON 1683). 1:50 7‐AAD (BD Biosciences, 559,925) was added to sort away dead cells and cells were selected for GFP with FACS performed on a FACSAria II Cell Sorter (BD Biosciences, San Jose, California, USA); 500 to 1000 GFP‐positive cells were plated per well in a 6‐well plate (Corning, 3516) in FACS medium. Until day 7, cells were expanded in FACS medium, thereafter in Stemflex medium (Life technologies, A3349401). When decent single‐cell colonies emerged, they were picked and transferred to one Geltrex‐coated (Life technologies, A1413202) well of a 12‐well plate (Corning, 3513) in Stemflex medium (Life technologies, A3349401) containing 5 μM Y‐27632 ROCK‐inhibitor (Axon Biochemicals, AXON 1683) for 24 h. As soon as clones reached 70%–80% confluency, part of the cells were harvested for genomic DNA extraction and the rest were cryopreserved as follows. To freeze iPSCs, cells were washed with PBS and incubated for 2 min in 0.5 mM EDTA. Cells were detached by spraying PBS or Stemflex medium (Life technologies, A3349401) on the cells and collected in a tube that was spun down for 4 min at 200×g. Supernatant was removed and pellets were resuspended in cold freezing medium containing FBS (Sigma‐Aldrich, F7524) and 10% DMSO, snap frozen at −80°C and stored in liquid nitrogen. To thaw iPSCs, cells were put in a warm water bath and when thawed pipetted into Stemflex medium (Life technologies, A3349401) and spun down for 4 min at 200×g. Supernatant was aspirated and pellets were dissolved in Stemflex (Life technologies, A3349401) containing 5 μM Y‐27632 ROCK‐inhibitor (Axon Biochemicals, AXON 1683) and plated in Geltrex‐coated (Life technologies, A1413202) dishes. The selected clone, where Sanger sequencing confirmed the indel leading to a premature termination codon, was tested for off‐target effects (Supplementary Table S3), expanded, and used to generate unguided neural organoids.

### 2.6 Immunohistochemistry

Neural organoids were fixed in 4% paraformaldehyde overnight at 4°C and washed three times for 10 min in PBS before being incubated overnight at 4°C in 30% sucrose in PBS. Subsequently, neural organoids were embedded in Tissue‐Tek(R) O.C.T. Compound (Sakura Finetek, 4583), snap‐frozen in a dry ice/ethanol slurry and stored at −80°C until further use. Sections of 15 μm thickness were obtained with a.

Leica CM1950 cryostat (Leica Biosystems, Illinois, USA), collected on SuperFrost(R) PLUS (VWR, 631–0108) slides and stored at −80°C until further processing. For immunohistochemistry, sections were blocked for 1 h at room temperature in blocking buffer consisting of 10% normal donkey serum (Jackson ImmunoResearch, 017–000‐121), 3% BSA (Sigma‐Aldrich, A4503‐100) and 0.1% Triton‐X (Sigma‐Aldrich, T8787‐100) in PBS. Next, samples were incubated with primary antibodies in blocking buffer overnight at 4°C. Slides were then washed three times for 10 min in PBS containing 0.05% Tween 20 (Merck, 817,072) (PBS‐T) before being incubated with secondary antibodies and Hoechst (Sigma‐Aldrich, 94,403) in blocking buffer for 1 h at room temperature. Then, slides were washed three times for 10 min in PBS‐T. The antibodies that were used in the current study can be found in Table S4. Finally, samples were mounted on glass coverslips using Fluorosave (CalBioChem, 345,789) and imaged using a Zeiss Axioscope A1 (Zeiss, Oberkochen, Germany) and a Zeiss LSM 880 confocal microscope (Zeiss, Oberkochen, Germany).

### 2.7 Quantification of Immunofluorescent Microscopy Images

Multiple immunofluorescent microscopy images of Hoechst, PAX6, and SOX2 from sliced 9‐day‐old organoids obtained with a Zeiss Axioscope A1 were processed and analyzed as follows. Using ImageJ, background was subtracted (rolling ball radius 30), where after a threshold was set. Images were masked using the “make binary” function. Next, the “watershed” function was applied to improve boundaries between different nuclei. Using “Analyze particles”, signal was quantified with a minimum particle size of 5μm^2^, yielding data points for the number of positive nuclei, as well as the total signal area. To obtain PAX6 and SOX2 signal relative to the number of cells, we applied the following formula: total SOX2 or PAX6 area/number of nuclei. One‐way ANOVA with multiple testing correction was performed using GraphPad software.

### 2.8 RNA Isolation and cDNA Synthesis

For RNA isolation, five or more neural organoids were pooled and medium was removed carefully. iPSCs that reached 80% confluency were manually detached in PBS. Organoid and iPSC samples were homogenized in 1 mL of Qiazol (QIAGEN, 79306) with an ULTRA‐TURRAX(R) (IKA, 0003737000), followed by addition of chloroform in a 1:5 ratio to Qiazol and centrifugation at 12,000 × *g* at 4°C for 20 min. Then, the aqueous top phase was collected and mixed with 500 μL isopropanol and then stored overnight at −20°C to allow the RNA to precipitate. Subsequently, samples were centrifuged at 12,000 × *g* at 4°C for 30 min and the supernatant was aspirated. Pellets were washed three times with 75% ethanol, air‐dried, and dissolved in TE‐buffer (Invitrogen, 12,090–015). RNA concentration was measured using a Varioskan Flash (Thermo Scientific, N06354) or NanoDrop (ThermoFisher Scientific, ND‐2000) and cDNA was synthesized using a Quantitect Reverse Transcription kit (QIAGEN, 205311) as follows. After removal of potential genomic DNA contamination using gDNA wipe‐out buffer from the kit, 500 ng of RNA was reverse transcribed at 42°C for 30 min followed by incubation at 95°C for 3 min to deactivate the RT enzyme. Samples were diluted 1:20 in RNAse‐free water and stored at −20°C.

### 2.9 RT‐qPCR

Reverse transcription quantitave PCR (RT‐qPCR) was performed on the QuantStudio 6 Flex Real‐Time PCR System (ThermoFisher Scientific Inc.) using a 384‐well plate under the following conditions: denaturing at 95°C for 10 min, 40 cycles with 95°C for 15 s and annealing at 60°C for 1 min, followed by a dissociation stage where the temperature was increased from 60°C to 95°C. Per reaction, 5 μL FastStart Universal SYBR Green Master (Roche, 04913914001) 3 μL MQ (Millipore, SYNS00000), 1 μL cDNA (RNA input concentration 2.5 ng/μL), and 1 μL 0.5 μmol/mL forward and reverse primer mix were used. Primers are listed in Table S1. Reactions were run in triplicates. Melting curve analysis was performed as a quality control. Gene expression was normalized to housekeeping genes *GAPDH*, *β‐Actin, TBP*, *SDHA*, and *RPII*, and visualized as 2^−ΔCt^.

### 2.10 Bulk RNA Sequencing

Organoids were pooled and RNA was extracted as described above. Sequencing libraries were prepared from polyadenylated RNA with QuantSeq 3′ mRNA Seq Library Prep Kit FWD for Illumina (Lexogen) with i5 and i7 dual indexing (for the data that are shown in Figure 2) or i7 single indexing (for the data that are shown in Figures 4 and 5). Concentration and quality of the libraries were measured using Qubit dsDNA HS Assay Kit (Invitrogen) and Fragment Analyzer HS NGS Fragment Kit (#DNF‐474, Agilent). Equimolar pooling was done to adjust to a final concentration of 22 nM (for the data that are shown in Figure 2) and 60 nM (for the data that are shown in Figures 4 and 5). Illumina NovaSeq 6000 and NextSeq 500 were used for sequencing in single‐end mode with 114 bp (for the data that are shown in Figure 2) and 86 bp‐long reads (for the data that are shown in Figures 4 and 5), respectively.

Reads without UMIs and “TATA” spacer were used further in analysis (removal with umi_tools 1.0.1 (Smith et al. 2017)). Quality control of the sequencing data was performed using FastQC (0.11.9 (Andrews et al. 2019)) and FastQ Screen (0.11.1 (Wingett and Andrews 2018)). Illumina adapters, indices, and low‐quality reads were removed with TrimmomaticSE (0.36 (Bolger et al. 2014)). Ribosomal and mitochondrial reads were removed using SortMeRNA (2.1b (Kopylova et al. 2012)). Remaining reads were aligned to human genome ( *Homo sapiens* GRCh38.87) using STAR (2.7.0f (Dobin et al. 2013)). Count matrices were generated using htseq‐count (0.11.4 (Anders et al. 2015)). ENSEMBL‐IDs were used to identify transcripts.

Data were further analyzed with R programming language (4.2.2 (R Core Team (2022))). Rlog transformed data were used in PCA. Org.Hs.eg.db (3.15.0 (Carlson 2019)) database was used for gene symbol conversion. Differential gene expression was analyzed using DESeq2 (1.36 (Love et al. 2014)) package. DEGs were identified using the following parameters: baseMean > 10, *p*_adj_ < 0.05, |log_2_FC| > 1. Gene Ontology (Carbon et al. 2021) enrichment analysis was performed using clusterProfiler (4.4.4 (Wu et al. 2021)) package, using function *enrichGO(keyType = “ENSEMBL”, ont = “ALL”, pAdjustMethod = “fdr”, pvalueCutoff = 0.1, minGSSize = 3)*. All genes in the dataset comprised the parameter *universe*.

### 2.11 Western Blot

For protein isolation, five or more neural organoids were pooled and medium was removed carefully. iPSCs that reached 80% confluency were manually detached in PBS. Organoid and iPSC samples were lysed in lysis buffer, consisting of 100 mM Tris (pH = 8.0), 140 mM NaCl, 20 mM EDTA, and 5% SDS, with 1:50 phosphatase inhibitor (Sigma‐Aldrich, P5726) and 1:25 protease inhibitor (Roche, 11,697,498,001), and homogenized with Ultra‐Turrax (IKA, 0003737000) for protein isolation. Protein concentrations were measured with PierceTM BCA Protein Assay Kit (Thermo Fisher, 23,225). Loading buffer containing 100 mM Tris (pH 6.8), 4% SDS, 20% glycerol and 10% 2 M Dithiothreitol (DTT; Sigma‐Aldrich, D9779) was added in a 1:1 ratio with lysis buffer, and samples were incubated for 10 min at 100°C for protein denaturation. To break down DNA, protein samples were threaded through a 25G syringe. Samples were stored at −20°C until further use. For western blotting, protein samples were stained with NuPAGE 4× LDS Sample Buffer (Invitrogen, NP0007) and 20–50 μg protein per lane was loaded and separated on a 10% SDS polyacrylamide gel with electrophoresis. The proteins were transferred onto a 0.45 μm Protran nitrocellulose membrane (GE Healthcare, A20485269) by wet blotting for 1h at 100 V. Revert 700 Total Protein Stain (LI‐COR), according to the manufacturer's protocol, was performed and visualized with the Odyssey CLx LI‐COR scanner. After removal of the remaining total protein stain, blots were blocked in Supermix blocking buffer, containing 25 mM Tris pH 7.4, 77 mM NaCl, 69 mM gelatin and 0.25% Triton X‐100, for 10–20 min at room temperature. Subsequently, the blots were incubated overnight with primary antibodies diluted in Supermix. The following day, blots were washed trice in TBS‐T with 1% Tween20 (Merck, 817,072) for 10 min and incubated with secondary antibodies anti‐rabbit IRDye800 or anti‐chicken AF647 for 1 h at room temperature. After three washes with TBS‐T and one with distilled water, blots were imaged with the Odyssey CLx LI‐COR scanner. Hereafter, images were analyzed in ImageJ software and LUT was inverted to visualize the blot background as white and the blotted protein as black, as follows. Image ➔ color ➔ gray. Image ➔ color ➔ invert LUT. For Figure S5A, the red channel showed the protein of interest and not the ladder; vice versa for the green channel. Therefore, for Figure S5A, we proceeded as follows. Image ➔ color ➔ split ➔ merge red/green channel ➔ LUT ➔ gray for both channels ➔ image ➔ type ➔ RGB color ➔ image ➔ type ➔ 32 bit ➔ invert LUT. These steps, along with the processed images are shown in Figure S9 (for Figure S2) and Figures S10 and S11 (for Figure S5).

### 2.12 PCR and Sanger Sequencing

PCRs were conducted using a T100 Thermal Cycler (Bio‐Rad, 1,861,096) with the following cycle conditions: 95°C for 5 min, 95°C for 30 s, 55°C–60°C for 30 s, 72°C for 1 min per 1000 base pairs of amplicon, for 36 (DNA) or 40 (cDNA) cycles followed by 10 min at 72°C. The exact annealing temperature for each primer can be found in Table S2. The total volume of the samples was 20 μL, consisting of 4 μL 5 × FIREPol master mix (Solis BioDyne, 04–12‐00125), 1 μL primer mix (0.5 μmol/mL forward/0.5 μmol/mL reverse in 1:10 ratio to MQ (Millipore, SYNS00000); primer specification see Supplementary Table S2), and cDNA of 12.5 ng RNA input or around 150–200 ng DNA in 15 μL MQ (Millipore, SYNS00000). PCR products were run on a 1% agarose gel at 80 V for around 1 h. Bands at the expected amplicon were cut out and DNA was extracted using a Purelink Quick Gel Extraction kit (Invitrogen, K210012) according to the manufacturer's protocol. The samples were dissolved in a gel solubilization buffer (L3, Invitrogen, 80021013a) and incubated at 50°C for around 10 min. Subsequently, 1 gel volume of isopropanol was added, and DNA was purified using a centrifuge. It was then eluted with 30 μL elution buffer (E5, Invitrogen, 8,005,075). Samples were sent for Sanger sequencing (Macrogen, Amsterdam, The Netherlands) with 0.5 μmol/mL primer in a 1:10 ratio to the sample.

## 3 Results

### 3.1 Mutant *GFAP* Disrupts Neuroectoderm Development of Neural Organoids

To study the effects of mutant GFAP on human brain development, we first generated unguided neural organoids (UNOs) from two AxD patient‐derived iPSC lines carrying a heterozygous R239C mutation in *GFAP*, as well as from a CRISPR/Cas9‐corrected isogenic control line (Battaglia et al. 2019; Li et al. 2018). Both AxD lines, hereafter referred to as R239C‐AxD‐1 (Battaglia et al. 2019) and R239C‐AxD‐2 (Li et al. 2018), were derived in two different laboratories from the fibroblasts of the same six‐year‐old male, early‐onset AxD patient who, following a period of clinical symptoms starting in the first year of life, died at the age of six. The isogenic control line was generated from the R239C‐AxD‐1 line and will hereafter be referred to as R239R‐Ctrl. We confirmed the mutations in both AxD lines and the correction of the mutation in the R239R‐Ctrl line using Sanger sequencing (Figure S1).

**FIGURE 1:**
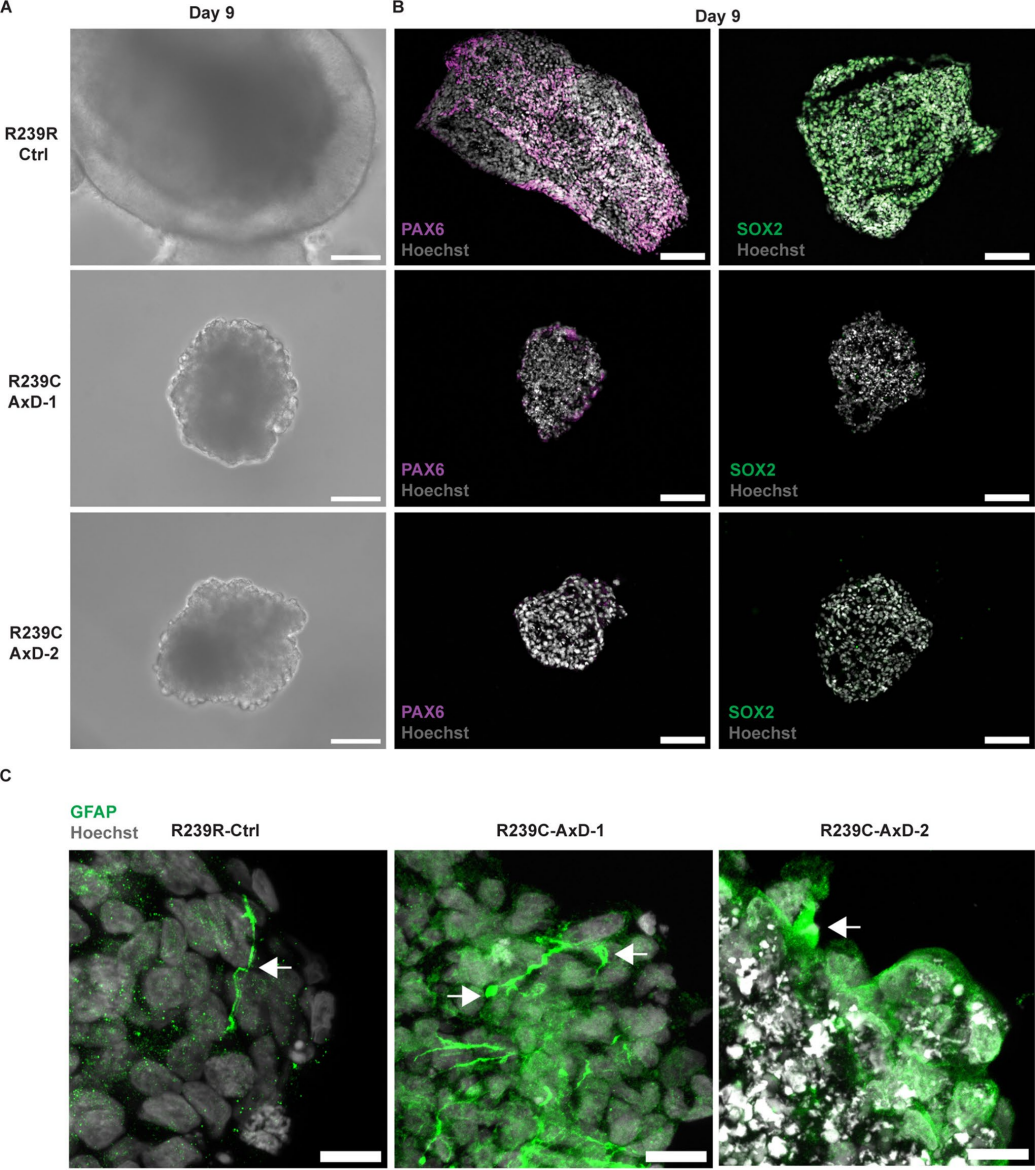
Neural organoids carrying mutant *GFAP* display neuroectodermal acquisition defects. (A) Brightfield microscopy images reveal strong morphological abnormalities in unguided neural organoids carrying mutant *GFAP* compared to isogenic control R239R‐Ctrl organoids. Top image: Arrow indicates expanding neuroepithelium. Middle and lower images: Arrow indicates bubble‐like protrusion in AxD organoids. Scale bars = 100 μm. (B) Immunofluorescent microscopy images for PAX6 (left column) and SOX2 (right column) indicate decreased expression of PAX6 and SOX2 in AxD organoids compared to isogenic control R239R‐Ctrl organoids. Scale bars = 100 μm. (C) Immunofluorescent microscopy images for GFAP in 9‐day‐old unguided neural organoids reveal GFAP^+^ filament like structures in R239R‐Ctrl, as well as aggregate‐like structures in AxD organoids, as indicated by arrows. Scale bars = 25 μm. Hoechst stains nuclei in all panels.

We observed that, 9 days after the generation of embryoid bodies (EBs), using the Aggrewell800 plate, UNOs derived from both AxD lines exhibited severe morphological abnormalities when compared to R239R‐Ctrl UNOs (Figure 1A). This was characterized by bubble‐like protrusions on the edges of the AxD UNOs, starkly contrasting the expanding neuroepithelium of the R239R‐Ctrl UNOs that is reminiscent of typical neural organoid development (Lancaster et al. 2013). To investigate whether this abnormal morphology paralleled abnormal expression of developmental markers, we performed immunohistochemistry for the neuroectoderm marker PAX6 and the neuroectoderm/pluripotency marker SOX2 at day 9. Whereas R239R‐Ctrl UNOs showed an abundance of PAX6^+^ cells and SOX2^+^ cells, AxD UNOs failed to acquire notable PAX6 and SOX2 expression (Figure 1B). Quantification of the immunofluorescent signal confirmed a significantly lower amount of PAX6 and SOX2 in AxD UNOs compared to R239R‐Ctrl UNOs (Figure S2D).

Because of this very early developmental phenotype, we reasoned that mutant GFAP had to be expressed very early in UNO development, which is in contrast to the assumption that GFAP is only expressed at later stages, when glial commitment has occurred. Interestingly, immunohistochemistry revealed the presence of GFAP^+^ structures in 9‐day‐old UNOs derived from both AxD lines, as well as from the R239R‐Ctrl line (Figure 1C). Notably, whereas R239R‐Ctrl UNOs showed sparse GFAP^+^ filament‐like structures, the GFAP in AxD UNOs was typically organized in aggregate‐like structures, reminiscent of the hallmark Rosenthal fibers (RFs) observed in AxD patient astrocytes (Heaven et al. 2016; Sosunov et al. 2017).

These results suggest that the presence of an R239C mutation in *GFAP* hampers neural organoid development.

### 3.2 GFAP KO in the R239C‐AxD‐2 iPSC Line Partially Rescues Neural Organoid Development

In order to demonstrate that GFAP was causative of the developmental phenotype in AxD UNOs, we knocked out GFAP in the R239C‐AxD‐2 line by guiding Cas9 to exon 1 of *GFAP* to generate a frameshift‐induced premature termination codon leading to subsequent nonsense‐mediated RNA decay. Sanger sequencing of this newly generated AxD‐GFAP‐KO iPSC line revealed a 25‐dbp deletion in exon 1 of *GFAP*, resulting in a premature termination codon (Figure S2A). Importantly, the potential off‐target genes *HLA‐F*, *NFIC*, and *RBFOX1* were unaltered compared to the parental R239C‐AxD‐2 line (Figure S6). We did observe two heterozygous intronic mutations in the *FABP6* gene of the AxD‐GFAP‐KO iPSC line but regarded this as not important due to the fact that they were intronic (Figure S6). UNOs generated from this iPSC line showed a clear rescue of the morphological phenotype that we observed in AxD UNOs. At day 9, R239C‐AxD‐KO UNOs were characterized by a round morphology similar to the earlier EB stage (Figure S2B) and presented with an abundance of SOX2^+^ cells (Figure S2C), indicating the presence of pluripotent cells. However, these organoids still largely lacked PAX6^+^ cells (Figure S2C). Quantification of immunofluorescent signal indeed revealed significantly less PAX6 signal in the AxD‐GFAP‐KO UNOs compared to R239R‐Ctrl (Figure S2D), suggesting that neural lineage commitment of R239C‐GFAP‐KO was not fully rescued. Western blot analysis confirmed the presence of GFAP in R239R‐Ctrl, R239C‐AxD‐1, and R239C‐AxD‐2, and the loss of GFAP in R239C‐AxD‐KO UNOs (Figure S2E,F).

These results further suggest that mutant GFAP is important for inducing neuroectodermal acquisition defects in UNOs, but that knocking out GFAP only partially rescues this phenotype.

### 3.3 Altered Lineage Commitment of Neural Organoids Carrying Mutant *GFAP*

**FIGURE 2:**
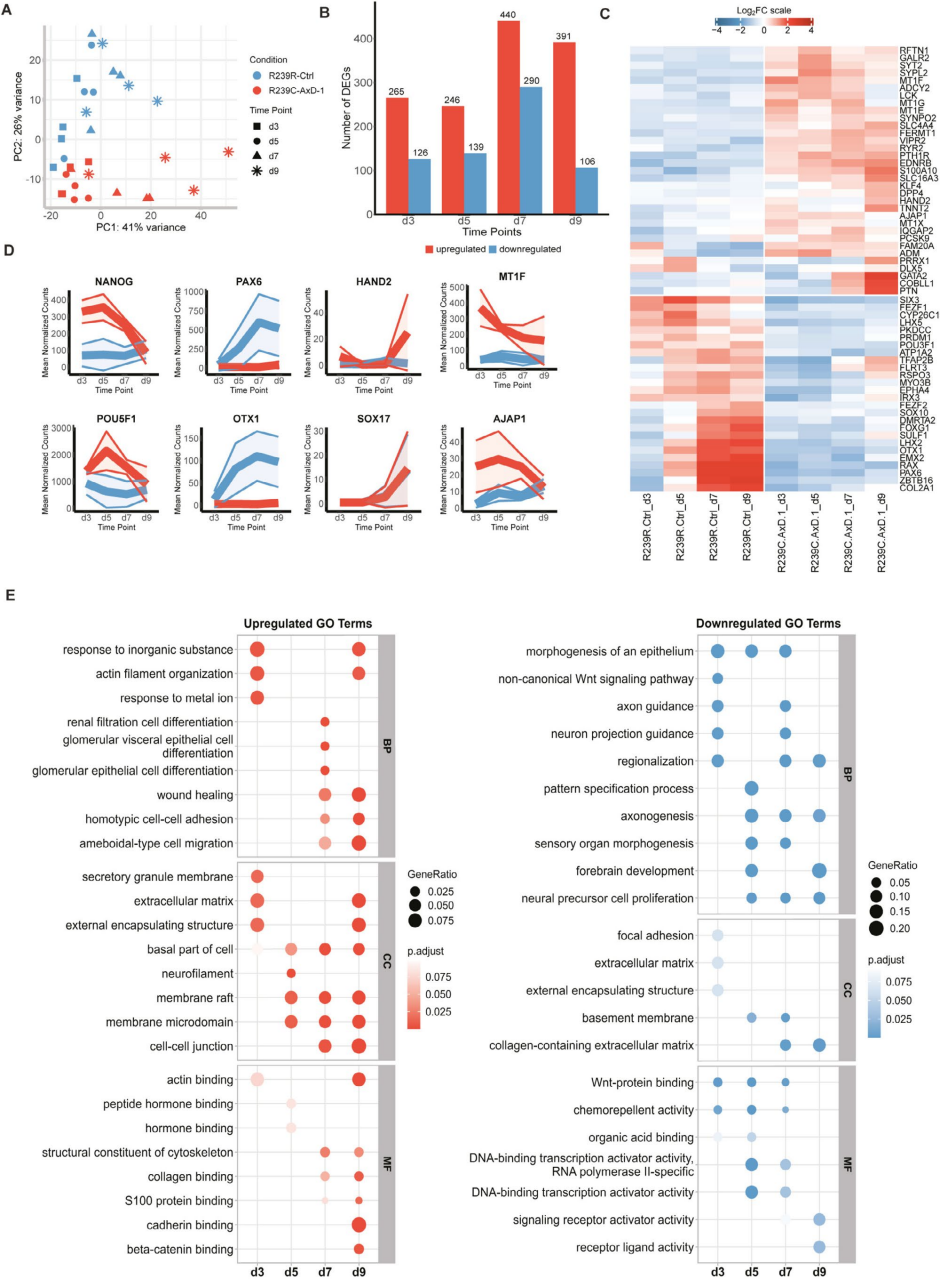
Bulk RNA sequencing reveals severely altered lineage commitment of AxD organoids. (A) PCA plot showing R239R‐Ctrl and R239C‐AxD‐1 neural organoids at different timepoints. (B) Bar graph showing the number of DEGs between R239R‐Ctrl and R239C‐AxD‐1 neural organoids at all timepoints. |log_2_FC| > 1, *P*_adj_ < 0.05. (C) Heatmap plot revealing lack of expression of neural commitment markers in R239C‐AxD1 neural organoids compared to R239R‐Ctrl, and an increase in mesodermal and stress markers. |log_2_FC| > 1, *p*_adj_ < 0.05. (D) Timeline plots showing expression over time of various developmental markers for R239R‐Ctrl and R239C‐AxD‐1 neural organoids. (E) Plot showing upregulated and downregulated GO terms in R239C‐AxD‐1 neural organoids compared with R239R‐Ctrl, over time. BP = biological processes, CC = cellular component, MF = molecular function. |log_2_FC| > 1, *p*_adj_ < 0.05. *N* = 4 independent batches of organoids.

To gain insight into the gene expression profiles underlying this defect in neuroectoderm commitment, we performed bulk RNA sequencing on 3‐, 5‐, 7‐, and 9‐day‐old R239C‐AxD‐1 and R239R‐Ctrl UNOs. Such a timeline, we hypothesized, would yield insight into the very early lineage commitment of EBs. The initial principal component analysis (PCA) revealed that, at all timepoints, AxD UNOs clustered separately from Ctrl UNOs and that difference increased over time (Figure 2A). Next, we performed a differential expression analysis (DEA) using DESeq2 and identified 391, 385, 730 and 497 differentially expressed genes (DEGs) in 3‐, 5‐, 7‐ and 9‐day‐old UNOs, respectively (Figure 2B). DEA revealed a significant downregulation of key neurogenesis markers such as *PAX6*, *EMX2*, *OTX2*, and *SIX3* in R239C‐AxD‐1 UNOs, whereas R239R‐Ctrl organoids gained expression of these markers over time (Figure 2C). On the other hand, R239C‐AxD‐1 UNOs upregulated mesodermal transcription factors *GATA2*, *PRRX1*, and *HAND2*, cardiomyocyte‐specific *TNNT2*, as well as embryonic mesoderm ventralizing factor *BMP4*. These data together suggest commitment to a more mesodermal, rather than a neuroectodermal developmental trajectory (Figure 2C). Timeline plots revealed that R239C‐AxD‐1 UNOs, compared to R239R‐Ctrl UNOs, showed higher expression of pluripotency markers *POU5F1* (*Oct‐4*) and *NANOG* at day 3, but gradually lost this expression over time (Figure 2D). The lower expression of these pluripotency markers paralleled a rapid gain of neuroectoderm markers *PAX6* and *OTX1* in R239R‐Ctrl UNOs. R239C‐AxD‐1 UNOs, on the other hand, gained mesodermal transcription factor *HAND2*, but did not show increased expression of the endoderm development marker *SOX17* compared to R239R‐Ctrl. Moreover, R239C‐AxD‐1 UNOs showed increased expression of several metallothionein genes and the cell–cell adhesion protein *AJAP1* (Figure 2C), especially in 3‐day‐old UNOs during early stages of EB formation (Figure 2D), suggesting an increased susceptibility to stress and an altered response to the mechanical signals during EB formation. Gene Ontology (Carbon et al. 2021) upregulated terms for 3‐day‐old R239C‐AxD‐1 UNOs pointed to stress‐related terms such as *response to inorganic substance*, *response to metal ion*, as well as *actin filament organization* (Figure 2E). Over time, these terms were accompanied by more cytoskeleton‐related terms such as *membrane raft*, *cell–cell junction*, and *cadherin binding*, again suggesting an altered cytoskeletal response. GO downregulated terms in 3‐day‐old R239C‐AxD‐1 UNOs included *morphogenesis of an epithelium*, *non‐canonical Wnt signaling pathway*, and *axon guidance* (Figure 2E), suggesting altered pathway signaling in R239C‐AxD‐1 UNOs. Over time, downregulated terms transitioned into *forebrain development* and *axonogenesis*, further illustrating the inability of R239C‐AxD‐1 organoids to commit to neural development.

Overall, these results show that AxD embryoid bodies fail to develop into neural organoids and exhibit altered germ layer specification that is accompanied by stress and alterations in mechanical signaling.

### 3.4 Reducing Forced Cell Aggregation During EB Formation, as Well as Dual SMAD Inhibition, Rescues Altered Lineage Commitment of AxD Organoids

Previously, it has been reported that AxD cells show increased mechanotransduction signaling (Wang et al. 2018). Given our observations that alterations in mechanical signaling parallel altered lineage commitment in R239C‐AxD‐1 UNOs (Figures 1 and 2), and the fact that mechanotransduction can steer lineage commitment of stem cells (Rammensee et al. 2017), we hypothesized that AxD UNOs are hypersensitive to the mechanical stress of EB formation, leading to altered lineage commitment. Supported by our RNAseq data that highlighted *membrane raft* and *cell–cell junction* (Figure 2E), we reasoned that the forced aggregation of cells inside the reversed pyramid‐shaped microwells of the Aggrewell800 plate caused alterations in cell–cell adhesion, leading to stress‐impaired differentiation of R239C‐AxD‐1 UNOs.

**FIGURE 3:**
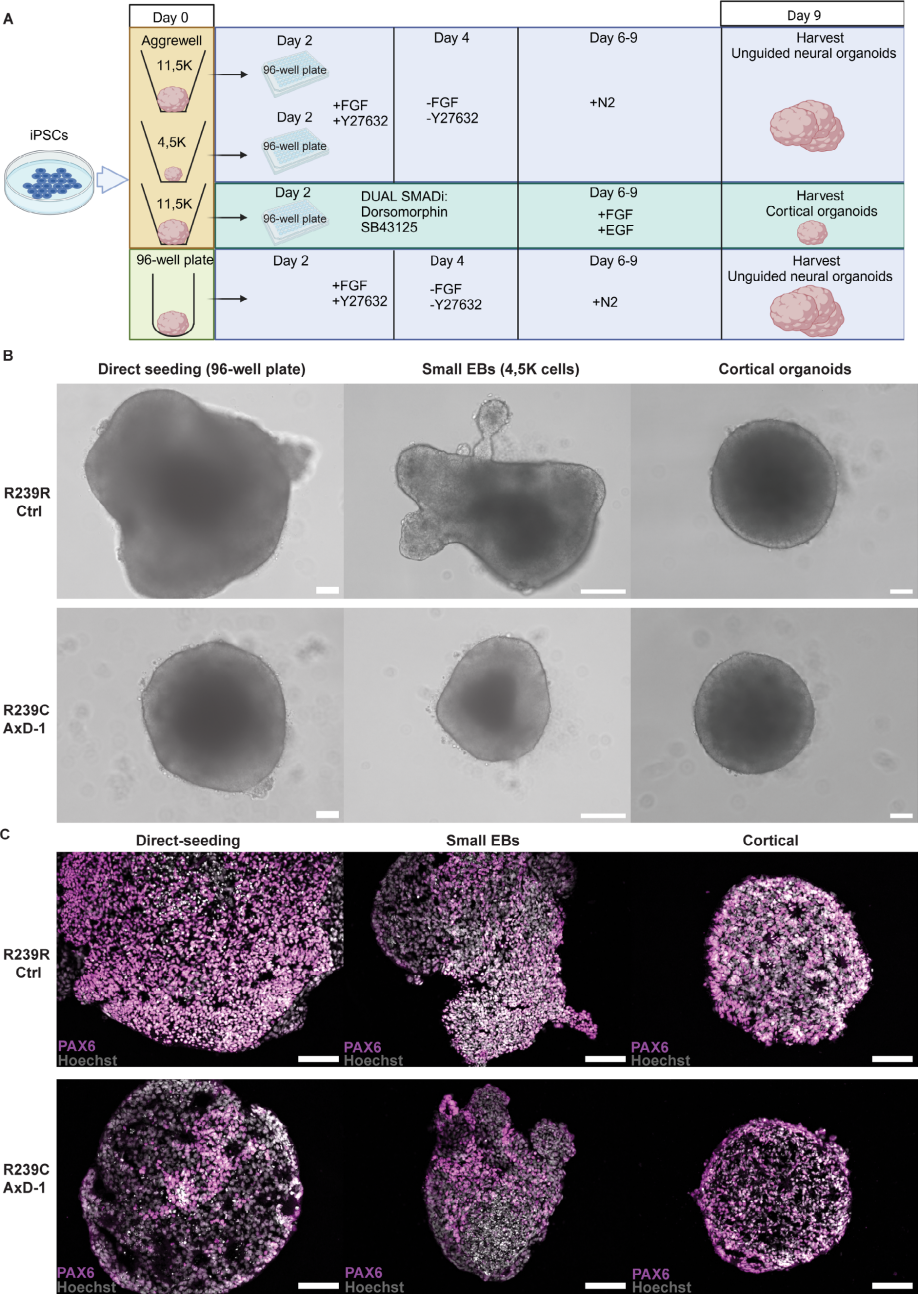
Prevention and rescue of neural commitment deficits in AxD neural organoids. (A) Protocols that were used to generate neural organoids in the prevention and rescue experiments. For full protocol description, see methods section. 3A was created with BioRender.com. (B) Brightfield microscopy images showing R239R‐Ctrl and R239C‐AxD‐1 neural organoids in prevention (left, middle) and rescue (right) experiments at day 9. (C) Representative immunofluorescent microscopy images revealing PAX6 expression for 9‐day‐old R239R‐Ctrl and R239C‐AxD‐1 neural organoids, from prevention (left, middle) and rescue (right) experiments. All nuclei were stained with Hoechst. Scale bars = 100 μm.

To investigate this, we took two approaches. First, we tried to prevent the differentiation phenotype by reducing the mechanical stress of EB formation, either by generating EBs directly in an ultra‐low attachment, round‐bottom 96‐well plate (direct‐seeding UNOs), or by reducing the starting number of iPSCs per EB from 11,500 to 4,500 (small UNOs) (Figure 3A). Second, we guided the differentiation of EBs into cortical organoids (Yoon et al. 2019) (CORs) using dual SMAD inhibition to force neuroectoderm commitment of Aggrewell800‐generated EBs.

Interestingly, at day 9, R239C‐AxD‐1 direct‐seeding UNOs had not acquired a protruding, expanding neuroectoderm (Figure 3B), similar to their Aggrewell800 counterparts that are hereafter referred to as confined UNOs (Figure 1A). However, R239C‐AxD‐1 direct‐seeding UNOs did not exhibit the severe morphological phenotype of the confined UNOs but retained their round, EB‐like morphology. R239R‐Ctrl direct‐seeding UNOs, on the other hand, generated expanding neuroepithelia (Figure 3B) similar to the confined UNOs (Figure 1A). This suggests that merely changing the method of EB formation has a profound effect on organoid development. Similarly, we observed that R239C‐AxD‐1 small UNOs were also able to prevent the severe morphological phenotype as shown on day 9 (Figure 3B). By applying dual SMAD inhibition in the CORs, we were able to rescue the morphological phenotype of R239C‐AxD‐1 UNOs, even when EBs were generated using the confining approach (Figure 3B). Immunohistochemistry for PAX6 and SOX2 of 9‐day‐old R239C‐AxD‐1 direct‐seeding UNOs, small UNOs, and CORs, as well as for their isogenic counterparts, revealed that both R239R‐Ctrl and R239C‐AxD‐1 organoids generated via these approaches were capable of acquiring PAX6^+^ cells (Figure 3C). This suggests that R239C‐AxD‐1 direct‐seeding UNOs, as well as small EB UNOs and CORs, do not exhibit the severe morphological phenotype of confined organoids and show the capacity to commit to the neuroectoderm lineage.

All in all, these results suggest that the mechanical properties of EB formation tightly interact with signaling pathways that steer neural lineage commitment and that R239C‐AxD‐1 cells show increased susceptibility to alterations in this cross‐signaling.

### 3.5 AxD Direct‐Seeding Neural Organoids Show Delayed Differentiation

Given these model‐specific phenotypes, we wanted to investigate the gene expression profiles underlying the specific contributions of the confining and direct‐seeding methods to both phenotypes. To do so, we generated R239C‐AxD‐1 and R239R‐Ctrl UNOs using the confining and direct‐seeding approach, from the same iPSC suspension, after which we again performed bulk RNAseq on 3‐, 5‐, 7‐, and 9‐day‐old UNOs.

**FIGURE 4:**
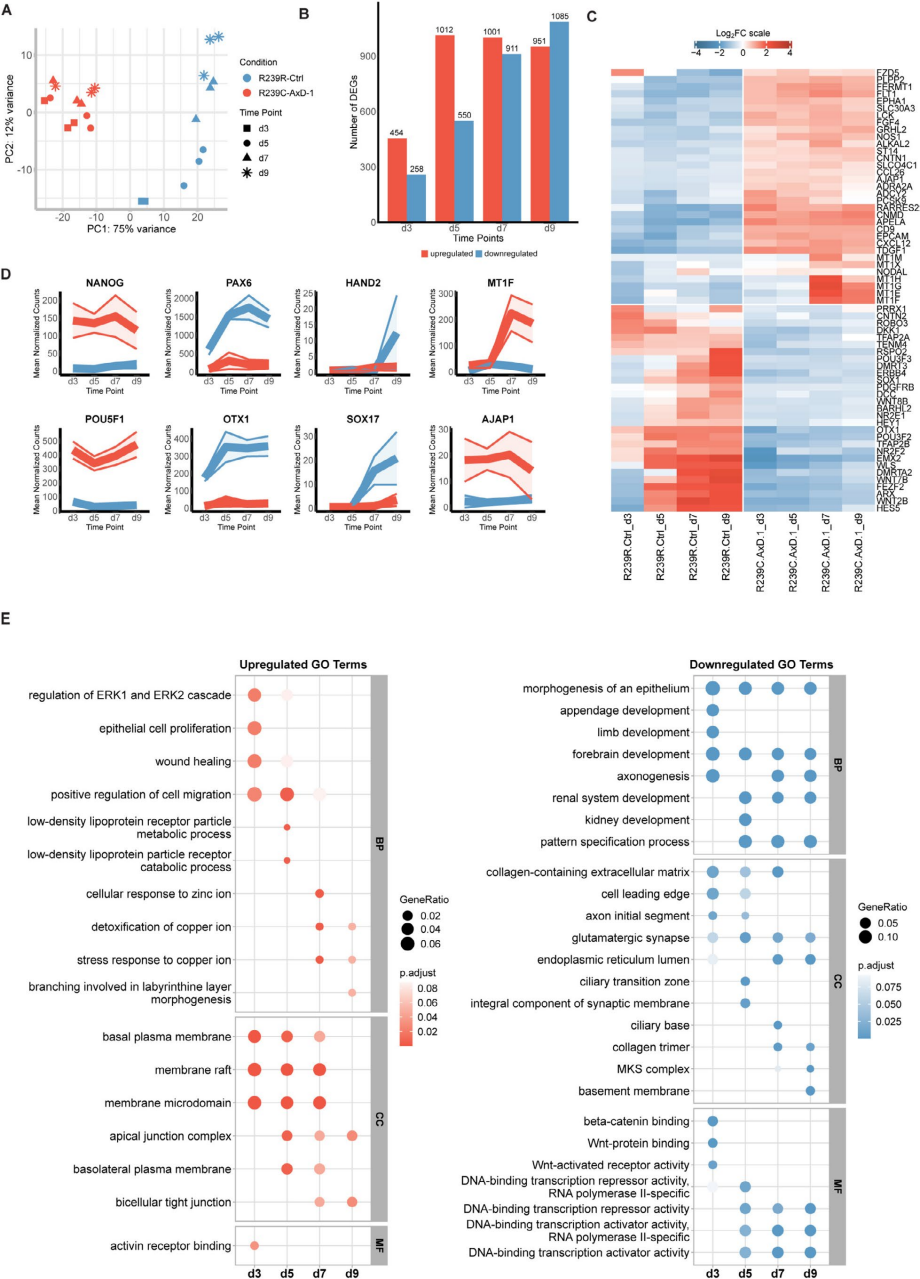
Bulk RNA sequencing reveals delayed lineage commitment of direct seeding AxD neural organoids. (A) PCA plot showing R239R‐Ctrl and R239C‐AxD‐1 neural organoids at different timepoints. (B) Bar graph showing the number of DEGs between R239R‐Ctrl and R239C‐AxD‐1 neural organoids at all timepoints. (C) Heatmap plot revealing lack of expression of neural commitment markers in R239C‐AxD1 neural organoids compared to R239R‐Ctrl, and an increase in stress markers. |log_2_FC| > 1, *p*_adj_ < 0.05. (D) Timeline plots showing expression over time of various developmental markers for R239R‐Ctrl and R239C‐AxD‐1 neural organoids. (E) Plot showing upregulated and downregulated GO terms in R239C‐AxD‐1 neural organoids compared with R239R‐Ctrl, over time. BP = biological processes, CC = cellular component, MF = molecular function.

First, we analyzed the RNAseq data of the direct‐seeding approach, again comparing AxD with Ctrl. PCA revealed that, as early as day 3, R239C‐AxD‐1 UNOs clustered separately from Ctrl UNOs (Figure 4A). Moreover, the R239C‐AxD‐1 clusters did not further separate over time, in stark contrast to Ctrl organoid clusters that continued to deviate from their origin over time. Next, we performed a DEA using DESeq2 and identified 712, 1562, 1912, and 2036 DEGs in 3‐, 5‐, 7, and 9‐day‐old R239C‐AxD‐1 UNOs, respectively (Figure 4B). Moreover, DEA revealed that R239C‐AxD‐1 direct‐seeding UNOs, similar to confined R239C‐AxD‐1 UNOs, failed to acquire expression of neuroectodermal *PAX6*, but retained higher expression of pluripotency genes *NANOG, SOX2*, and *POU5F1/OCT‐4* compared to the R239R‐Ctrl direct‐seeding organoids. On the other hand, R239R‐Ctrl direct‐seeding UNOs rapidly gained *PAX6* expression over time (Figure 4C,D). Moreover, similar to confined UNOs, R239C‐AxD‐1 direct‐seeding UNOs upregulated multiple metallothioneins as well as cell adhesion protein *AJAP1*. Timeline plots further illustrated that whereas R239R‐Ctrl UNOs lost expression of pluripotency markers *POU5F1*(Oct‐4) and *NANOG* over time, R239C‐AxD‐1 UNOs, retained this expression, suggesting a delayed differentiation of R239C‐AxD‐1 direct‐seeding UNOs. (Figure 4D). The loss of pluripotency again paralleled a rapid gain of neuroectoderm markers *PAX6* and *OTX1* in R239R‐Ctrl UNOs, whereas in, R239C‐AxD‐1 UNOs, this was paralleled by an increased expression of metallothioneins (Figure 4C,D), suggesting a response to stress in R239C‐AxD‐1 UNOs. This suggests that R239C‐AxD‐1 direct‐seeding UNOs fail to exit pluripotency, at least within the investigated timeframe, in order to commit to the neural lineage. RT‐qPCR analysis of *PAX6* and *SOX2* revealed that R239C‐AxD‐1 small UNOs, as well as CORs displayed a similar phenotype as the UNOs, namely a decreased loss of pluripotency marker *SOX2*, and reduced expression of *PAX6* (Figure S3). Downregulated GO terms for R239C‐AxD‐1 direct‐seeding UNOs included *forebrain development*, *axonogenesis*, and *pattern specification process*, as well as *beta‐catenin binding* and *Wnt protein binding* (Figure 4E). On the contrary, upregulated GO terms transitioned over time from *regulation of ERK1 and ERK2 cascade* to *stress response to copper iron* and *apical junction complex*.

These data suggest that R239C‐AxD‐1 direct‐seeding UNOs are delayed in neuroectodermal acquisition compared to their isogenic controls, also exhibit stress, as well as changes in cytoskeletal dynamics, but do not alter their lineage commitment towards a mesodermal fate, as did the confined R239C‐AxD‐1 UNOs.

### 3.6 AxD Neural Organoids Are More Sensitive to Aggrewell800 Induced Confinement

**FIGURE 5:**
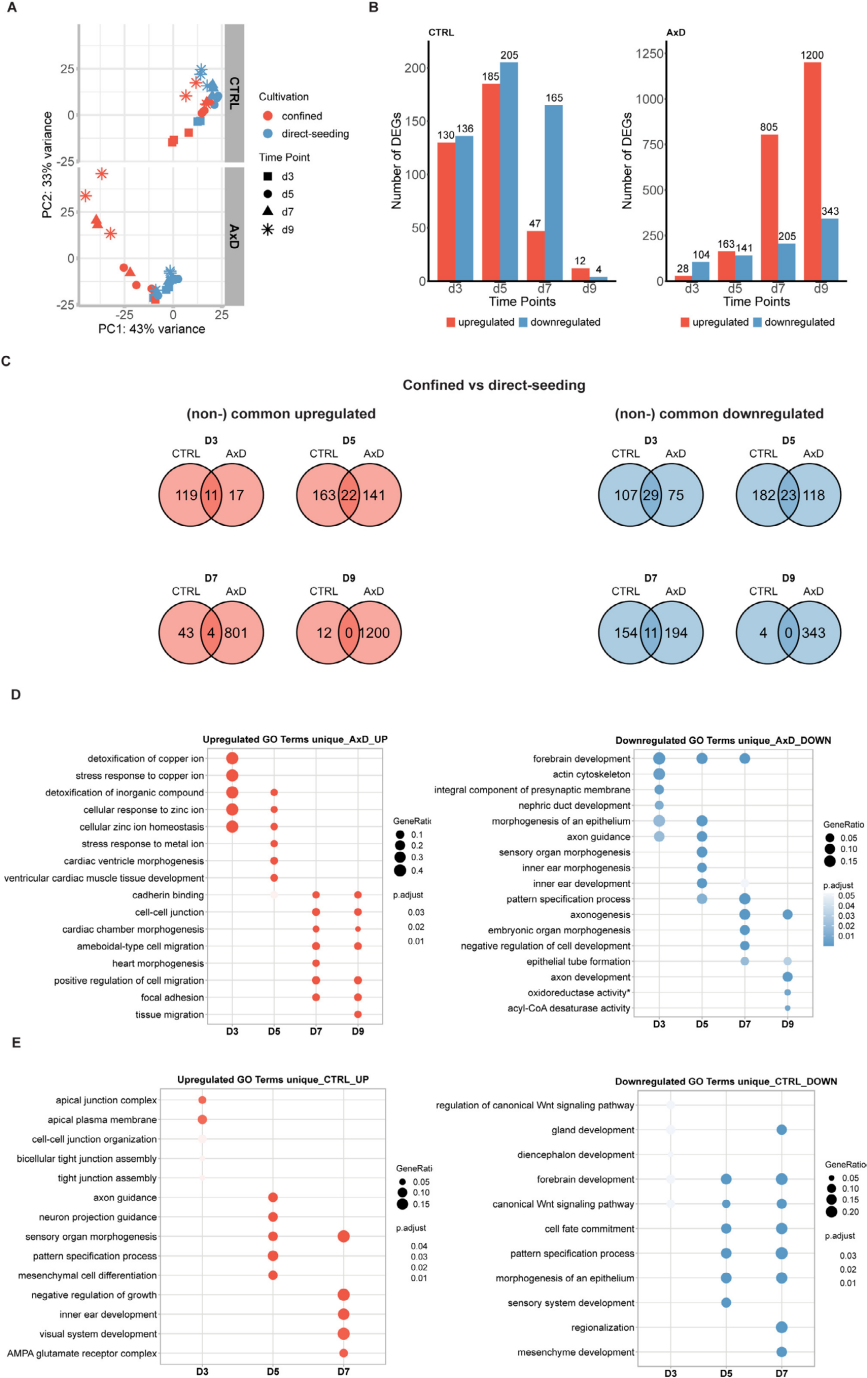
Comparison of confined and direct‐seeding unguided neural organoids using bulk RNA sequencing reveals an increased susceptibility to stress in AxD organoids. (A) PCA plot showing R239R‐Ctrl and R239C‐AxD‐1 neural organoids at different timepoints for the confined and direct‐seeding method. (B) Bar graphs showing the number of DEGs at day 3,5,7 and 9 between confined and direct seeding neural organoids for R239R‐Ctrl (left) and R239C‐AxD‐1 (right). *p* < 0.05, Log_2_FC −1,1. (C) Venn diagrams showing the common and non‐common DEGs between R239R‐Ctrl and R239C‐AxD‐1 neural organoids for the confined vs. direct seeding method comparison (red = up, blue = down in confined). (D) Up‐(left) and downregulated (right) GO terms for the confined compared to the direct seeding method that are unique for R239C‐AxD‐1 neural organoids compared to R239R‐Ctrl. (E) Up‐(left) and downregulated (right) GO terms for the confined compared to the direct seeding method that are unique for R239R‐Ctrl neural organoids compared to R239C‐AxD‐1. *Oxidoreductase activity, acting on paired donors, with oxidation of a pair of donors resulting in the reduction of molecular oxygen to two molecules of water.

Next, using the RNAseq data, we investigated the specific contribution of the confining and direct‐seeding protocols to the AxD phenotypes of altered lineage commitment and delayed lineage commitment, respectively. Interestingly, PCA revealed that whereas R239R‐Ctrl Aggrewell800 and direct seeding UNOs clustered together, R239C‐AxD‐1 Aggrewell800 UNOs deviated strongly from direct‐seeding UNOs over time (Figure 5A). This stark contrast between AxD and Ctrl was further reflected in the number of DEGs between Aggrewell800 and direct‐seeding UNOs over time (Figure 5B). Whereas the number of DEGs decreased over time for the R239R‐Ctrl UNOs, it increased over time for the R239C‐AxD‐1 UNOs. Moreover, R239R‐Ctrl UNOs upregulated 130 and downregulated 136 genes in the confining approach at day 3, whereas R239C‐AxD‐1 UNOs only upregulated 28 and downregulated 104. At day 9, this pattern completely reversed where R239R‐Ctrl UNOs upregulated 12 and downregulated 4 genes, compared to 1200 and 343 for R239C‐AxD‐1 UNOs. These results suggest that whereas R239C‐AxD UNOs seem more sensitive to the method of EB formation, R239R‐Ctrl UNOs respond with more DEGs at the early EB stages but manage to cope over time.

Next, we sought to identify common DEGs for the Aggrewell800 versus direct seeding approach between R239C‐AxD‐1 and R239R‐Ctrl, as well as unique DEGs for each condition, as we reasoned that this would yield insight into the AxD‐specific response to EB formation sensitivity. First, we identified common DEGs, as well as non‐common DEGs for R239C‐AxD‐1 and R239R‐Ctrl and plotted the number of such DEGs using Venn diagrams (Figure 5C). GO analysis of the common upregulated DEGs, which included metallothionein genes as well as inhibitors of differentiation *ID1* and *ID4*, highlighted *stress response to metal ion, detoxification of inorganic compound and cell–cell junction organization*. Common downregulated GO terms highlighted metabolic processes, *pattern specification process* and organ development (Figure S4). Upregulated GO terms that were unique to R239C‐AxD‐1 highlighted *stress response to metal ion*, several cardiac developmental terms and cytoskeleton related terms, as well as *cadherin binding* and *focal adhesion* (Figure 5D). Downregulated terms unique to R239C‐AxD‐1 highlighted *forebrain development, axon guidance*, and terms related to organ development (Figure 5D). On the contrary, upregulated GO terms unique to R239R‐Ctrl UNOs highlighted *cell–cell junction organization* as well as *axon guidance* and *negative regulation of growth* (Figure 5E). Downregulated GO terms unique to R239R‐Ctrl UNOs, on the other hand, highlighted *forebrain development*, *canonical Wnt signaling pathway*, and *cell fate commitment*.

These results suggest that whereas R239C‐AxD‐1 and R239R‐Ctrl UNOs share a common response to the confining EB formation method through upregulation of several metallothionein genes, R239C‐AxD‐1 UNOs appear to exhibit an aggravated response that results in complete misdifferentiation.

### 3.7 Multiple *GFAP* Mutations Disrupt Lineage Commitment of Unguided Neural Organoids, Which Can Be Rescued With Dual SMAD Inhibition

**FIGURE 6:**
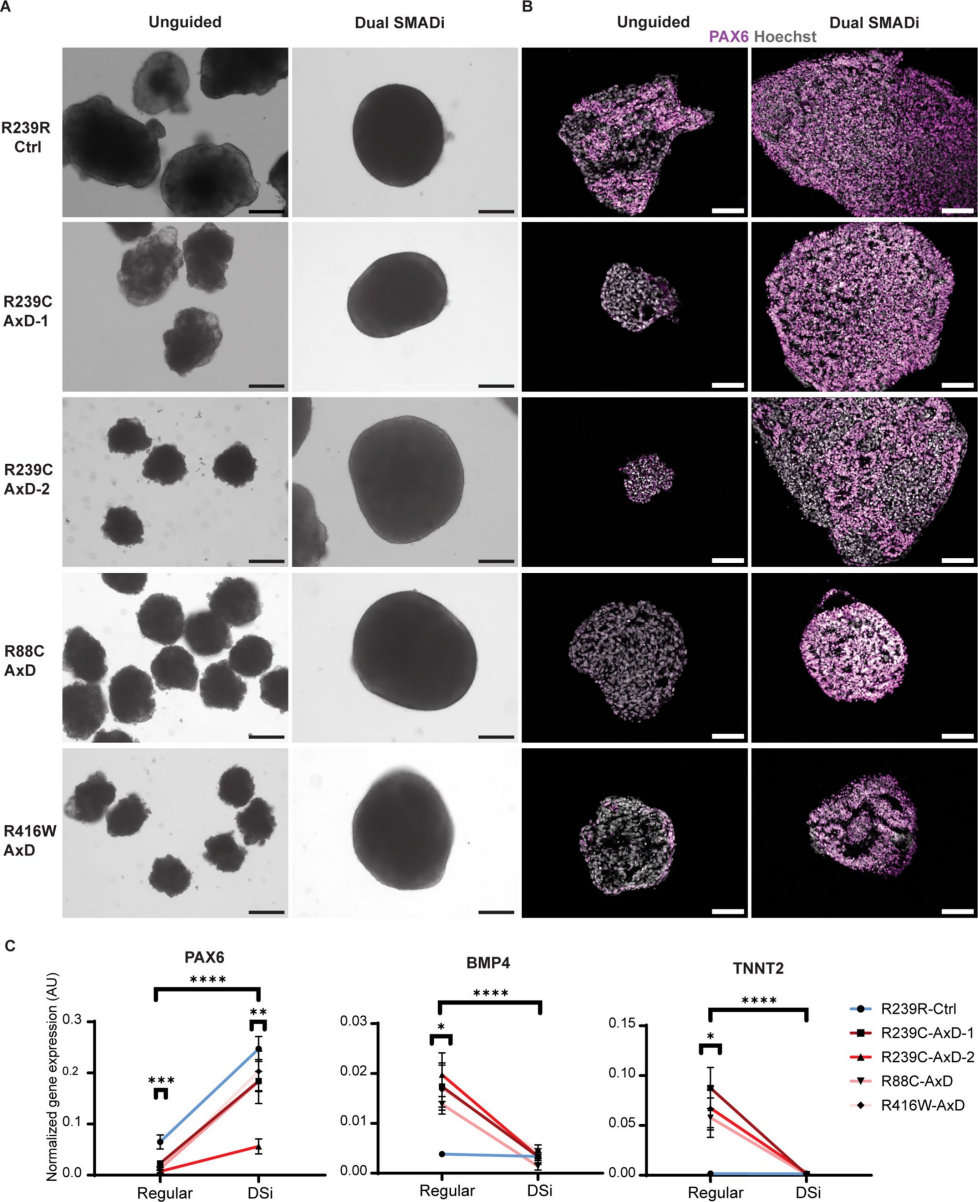
Multiple *GFAP* mutations disrupt lineage commitment of unguided neural organoids, which can be rescued with dual SMAD inhibition. (A) Brightfield microscopy images of 9‐day‐old R239C‐AxD‐1, R239C‐AxD‐2, R88C‐AxD, and R416W‐AxD unguided neural organoids (left) as well as organoids treated with dual SMAD inhibition (right). (B) Immunofluorescent microscopy images showing PAX6 (purple) and nuclei (gray) for 9‐day‐old R239C‐AxD‐1, R239C‐AxD‐2, R88C‐AxD, and R416W‐AxD unguided neural organoids (left) as well as organoids treated with dual SMAD inhibition (right). Scale bars = 100 μm. Hoechst stains nuclei in all images. (C) RT‐qPCR plots show expression of *PAX6* (left), *BMP4* (middle) and *TNNT2* (right) in regular as well as dual SMAD‐inhibited (DSi) R239C‐Ctrl, R239C‐AxD‐1, R239C‐AxD‐2, R88C‐AxD, and R416W‐AxD 9‐day‐old neural organoids. Data is shown as the mean ± SEM (*n* = 5 independent batches). Normalization was done to housekeeping genes *GAPDH, ACTB, TBP, SDHA, RPII*, and *18S*. One‐way ANOVA's were performed for comparison of conditions within treatment group (regular or DSi) and two‐way ANOVA's were performed to test for a general effect of treatment (regular or Dual SMAD inhibition (DSi)). *p < 0.05, **p < 0.01, ***p < 0.001, ****p < 0.0001.

Next, we investigated whether other AxD mutations in *GFAP* also alter lineage commitment of EBs. Therefore, we generated confined UNOs from AxD‐patient derived iPSCs carrying the heterozygous R88C (Jones et al. 2018) (R88C‐AxD) and R416W (Jones et al. 2018) (R416W‐AxD) mutations. Interestingly, when compared to R239R‐Ctrl, 9‐day‐old R88C‐AxD and R416W‐AxD UNOs exhibited similar morphological abnormalities that we observed previously for R239C‐AxD‐1 and R239C‐AxD‐2, characterized by bubble‐like protrusions on the organoid edges (Figure 6A). Immunohistochemistry for the neuroectoderm marker PAX6 again revealed a fair portion of PAX6^+^ cells (Figure 6B) in 9‐day‐old R239R‐Ctrl UNOs, but a significant decrease in PAX6 signal in R239C‐AxD‐1, R239C‐AxD‐2, R88C‐AxD, and R416W UNOs (Figure S7A), indicating a reduced commitment to the neural lineage. Western blot analysis revealed the presence of GFAP in these organoids (Figure S2D), as well as iPSCs from which they were derived (Figure S5). Since our cortical organoid protocol could prevent the morphological phenotype of R239C‐AxD‐1 UNOs (Figure 3B,C), we set out to determine whether dual SMAD inhibition could also rescue this phenotype in the R88C‐AxD, R416W‐AxD, and R239C‐AxD‐2 cell lines. Instead of using the cortical organoid protocol, we generated a combined cortical‐unguided organoid protocol, in which we added the same two SMAD inhibitors used in the cortical organoid protocol (dorsomorphin and SB431542) to the UNO protocol for the first 4 days of culture, when EBs are formed. The rest of the protocol was exactly the same. Using this approach would confirm that the observed rescue was really due to dual SMAD inhibition instead of altered medium composition between the COR and UNO protocols. R239R‐Ctrl iPSCs that were subjected to this protocol developed into dense‐looking, round‐shaped organoids (Figure 6A) that contained plenty of PAX6^+^ cells (Figure 6B). All AxD lines produced similar‐looking organoids (Figure 6A) that also contained a significant portion of PAX6^+^ cells (Figure 6B), indicating acquisition of neuroectodermal identity. RT‐qPCR revealed that all AxD lines produced UNOs with a significant reduction in *PAX6* expression (Figure 6C), a deficit that could be partially, but significantly, rescued with dual SMAD inhibition. Furthermore, *BMP4* was significantly upregulated in all AxD UNOs, as was *TNNT2*, suggesting commitment to a more mesoderm‐like fate in all AxD‐patient derived lines. Dual SMAD inhibition (DSi) strongly reduced *BMP4* and *TNNT2* expression in all AxD lines to a similar expression level as R239R‐Ctrl organoids. In addition, quantification of immunofluorescent signal for PAX6 revealed that DSi significantly rescued PAX6 expression in the AxD organoids (Figure S7B) so that it no longer differed significantly from R239R‐Ctrl organoids (Figure S7C).

These results suggest that multiple iPSC lines carrying various AxD mutations are prone to abnormal neural organoid development, which can be rescued by dual SMAD inhibition, indicating that AxD EBs generated with the Aggrewell800 system can commit to the neural lineage using forced neural induction, but not when differentiation is unguided.

### 3.8 GFAP Expression in Multiple AxD Patient Derived iPSC Lines, as Well as in a Healthy Control iPSC Line

**FIGURE 7:**
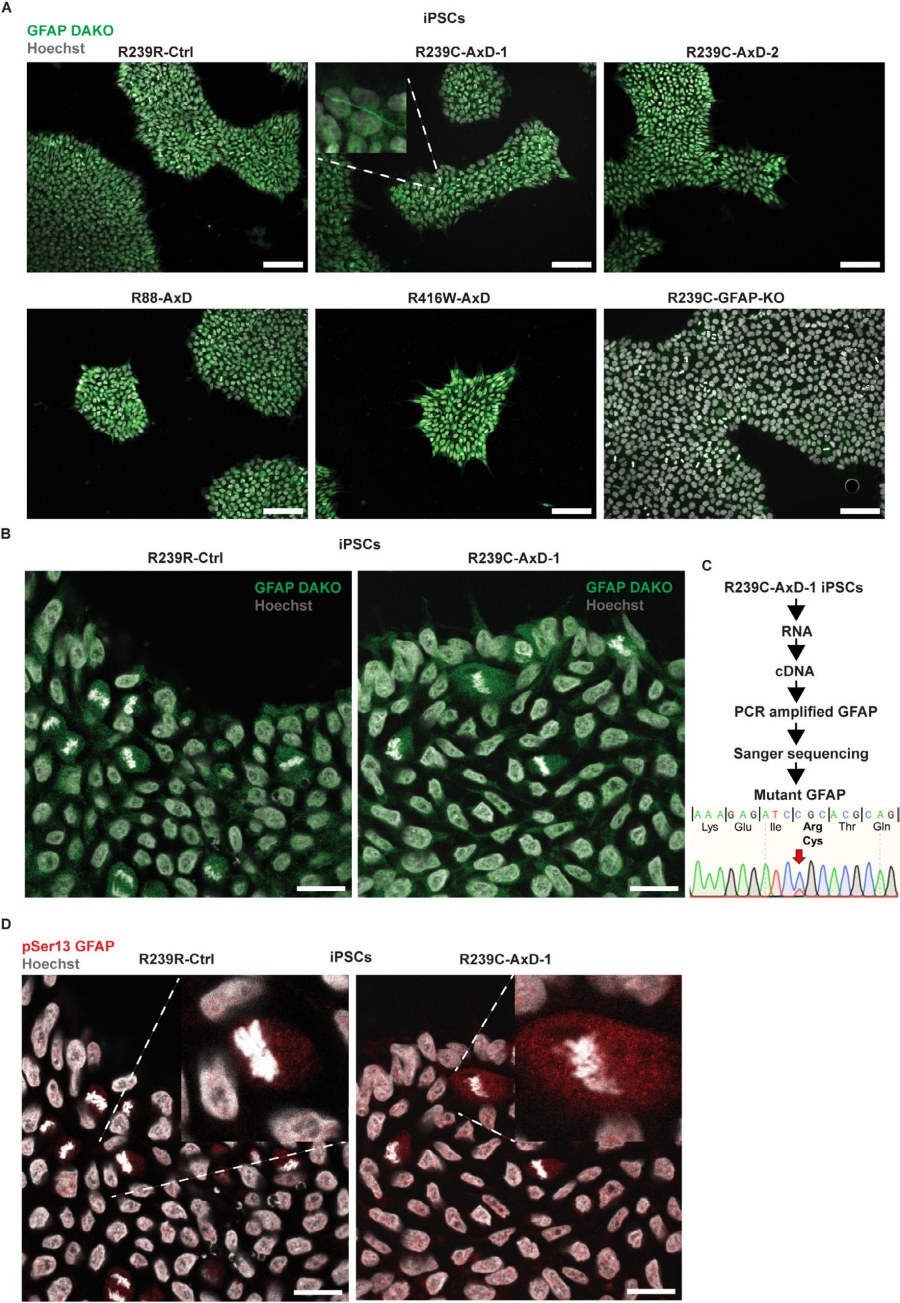
GFAP expression in iPSCs derived from multiple AxD patients. (A) Brightfield microscopy images of R239R‐Ctrl, R239C‐AxD‐1, R239C‐AxD‐2, R88C‐AxD, R416W‐AxD, and R239C‐GFAP‐KO iPSCs, showing GFAP expression using the GFAP DAKO antibody. Scale bars = 100 μm. (B) Confocal microscopy images for R239R‐Ctrl and R239C‐AxD‐1 iPSCs showing non‐filamentous GFAP. Scale bars = 20 μm. (C) Schematic showing the expression of mutant R239C *GFAP* cDNA in R239C‐AxD‐1 iPSCs. (D) Confocal microscopy images showing pSer13 GFAP being expressed in R239R‐Ctrl and R239C‐AxD‐1 iPSCs. Scale bars = 20 μm. Hoechst stains nuclei in all images.

The observation that GFAP is expressed early during organoid development made us wonder whether GFAP could already be expressed in iPSCs as well, given the severity and early onset of the phenotype in AxD UNOs. In order to explore this possibility, we performed immunocytochemistry for GFAP on R239R‐Ctrl, R239C‐AxD‐1, R239C‐AxD‐2, R88C‐AxD, R416W‐AxD, and R239C‐GFAP‐KO iPSCs. We observed a pattern of GFAP expression that appeared diffused throughout the cell, mostly in a non‐filamentous form (Figure 7A). Signal was also detected in healthy control iPSCs, whereas immunostaining with the secondary antibody alone did not show any signal, illustrating that the signal was due to the GFAP DAKO antibody (Figure S8A). Occasionally, however, GFAP^+^ filament‐like structures were observed (Figure 7A, top middle). To further characterize the GFAP expression in iPSCs, we performed confocal microscopy on R239R‐Ctrl and R239C‐AxD‐1 iPSCs, stained with the same GFAP DAKO antibody. This revealed to us that the signal for the GFAP DAKO antibody was mostly non‐filamentous and that the signal intensity was more obvious around the dividing chromosomes in mitotic cells (Figure 7B). To further investigate whether iPSCs can express *GFAP*, we isolated RNA from R239C‐AxD‐1 iPSCs, reverse transcribed it into cDNA, PCR‐amplified a region of *GFAP* that contained the R239C mutation, and sequenced the PCR product. Data analysis of the Sanger sequencing confirmed that the amplified product was *GFAP* and since no intronic regions were detected between multiple exons, it indicated that we amplified cDNA (Figure 7C). Interestingly, we noticed a heterozygous C → T mutation that would result in mutant R239C *GFAP* being heterozygously expressed (Figure 7C). Using western blot, we further assessed the expression of GFAP in iPSCs. We observed clear bands positive for the GFAP DAKO antibody at around 55 kDa, as well as for the GFAP SIGMA antibody at 50 kDa for all iPSC lines, including a healthy control iPSC line, as well as for 100‐day‐old healthy control UNOs derived from this healthy control iPSC line (Figure S5). As expected, we did not observe a matching band for the R239C‐GFAP‐KO line. Interestingly, for the GFAP DAKO antibody, we observed a slightly higher band for the iPSC lines derived from AxD patients, compared to the 100‐day‐old UNOs. When GFAP is phosphorylated on its head domain, it disassembles from its network, resulting in non‐filamentous GFAP (Kosako et al. 1997), a phenomenon that would be in line with the diffuse immunostaining signal that we observed (Figure 7A,B). Moreover, it was recently shown that GFAP is hyperphosphorylated at Ser13 in AxD patients and that AxD patient iPSC‐derived astrocytes exhibit phosphorylated Ser13‐GFAP (pSer13‐GFAP) (Battaglia et al. 2019).

In order to investigate whether pSer13‐GFAP was present in AxD patient‐derived iPSCs, we performed immunocytochemistry, using an antibody that recognizes pSer13‐GFAP, on R239R‐Ctrl and R239C‐AxD‐1 iPSCs. These are the same cell lines that were previously used to detect pSer13‐GFAP in AxD patient iPSC‐derived astrocytes (Battaglia et al. 2019). Interestingly, we observed a signal for the pSer13‐GFAP antibody in R239R‐Ctrl and R239C‐AxD‐1 iPSCs, where it presented as non‐filamentous and seemed to localize inside the nucleus, as shown using confocal microscopy (Figure 7D, Figure S8B). We also observed dividing iPSCs, in which the pSer13‐GFAP signal surrounded the mitotic spindle, in line with previous observations, where phosphorylated GFAP was shown to localize to the mitotic spindle during cell division of astroglial cells (Matsuoka et al. 1992). These results indicate that GFAP can be expressed in iPSCs.

## 4 Discussion

Here, we report that mutations in *GFAP* disrupt neural organoid development in a model‐specific manner. Using four AxD patient‐derived iPSC lines carrying mutations in the *GFAP* gene, we show that AxD unguided neural organoids (UNOs) generated at high cell density using an Aggrewell800 plate exhibit severe aberrations at a morphological, as well as cellular, level. These AxD organoids failed to commit to the neuroectoderm and altered their germline specification to favor mesoderm, which is in line with our previous findings (Matusova et al. 2025). This severe developmental phenotype could be reduced to a delayed differentiation by reducing cell density, generating EBs directly inside a 96‐well plate, or by forcing neuroectoderm commitment using cortical organoid protocols. This suggests that the combination of free choice of lineage commitment that characterizes unguided neural organoids (Lancaster et al. 2013; Pașca et al. 2022), combined with geometrical confinement, is required for the most severe phenotype. Removing one of these factors, either the free choice of differentiation through dual SMAD inhibition (Chambers et al. 2009) or the geometrically confining environment of the Aggrewell800 plate, reduces the phenotype to one that more resembles delayed lineage commitment, as opposed to faulty lineage commitment. Moreover, we showed that interfering with GFAP translation through a CRISPR/Cas9‐induced deletion in exon 1 of *GFAP* resulted in a rescue of the morphological phenotype that characterized the AxD Aggrewell800 UNOs.

Furthermore, the Aggrewell800 method also seemed to downregulate Wnt signaling in AxD as well as control organoids, but this pathway was also upregulated in AxD compared to control. The fine balance between TGF‐β/BMP and Wnt signaling is crucial for organ specification during embryogenesis (Tiedemann et al. 2001). Shifting the balance more towards Wnt with dual SMAD inhibition resulted in neuroectoderm formation. It remains, however, unknown how mutant GFAP makes embryoid bodies more prone to high‐cell density‐induced lineage commitment. TGF‐β/BMP4, as well as Wnt signaling, are closely intertwined with mechanical cues to guide lineage specification, where Wnt/β‐catenin signaling is a classic example of this intertwined relationship (Abuammah et al. 2018; Clevers and Nusse 2012). As such, aberrations in the interpretation of mechanical cues due to mutant GFAP could result in alterations of these signaling pathways. However, we cannot rule out that the bulk cell density per amount of medium volume affected our observed phenotype, since it has been shown to interact with pluripotent stem cell differentiation and TGF‐β signaling (Kempf et al. 2016).

Since in AxD, disease onset typically occurs after birth and can occur as late as adulthood (Messing 2018), it is unlikely that the severe developmental phenotype that we observed using the Aggrewell800 method resembles the pathogenesis of AxD as we currently know it. However, cell culture models might bring disease mechanisms to the surface more easily than would be observed in a living organism, either due to stressful conditions or because of the lack of compensatory mechanisms. If any of the phenotypes that we observed in AxD organoids were to resemble real‐life situations, it would most likely be delayed differentiation due to cellular stress, a known trigger to alter cell cycle progression and exit (Bhaduri et al. 2020; Rajasekaran et al. 2020; Schirmer et al. 2019; Spaas et al. 2021). Regardless, our data show that mutant *GFAP* can have profound effects on early organoid development. This clearly highlights the importance of cytoskeletal dynamics during early development and adds to the data that showed the importance of intermediate filaments during embryogenesis (Lim et al. 2020).

Furthermore, we observed a signal for multiple GFAP antibodies in iPSCs, a phenomenon that had not yet been described. The presence of GFAP in iPSCs allows for speculation that GFAP serves currently unknown functions. Of course, it remains to be investigated whether this is a cell culture artifact or whether GFAP is truly expressed in human embryonic stem cells in vivo. In support of our findings, using the OvoGrowth human gastrulation embryo database (ovogrowth.net) we found that GFAP expression was detected in some cells of the epiblast (Tyser et al. 2021), an embryonic cell mass that develops into the three primary germ layers (Rossant and Tam 2009). However, since *GFAP* expression during early human embryonic development has not been reported before, possibly due to the low mRNA levels that are ambiguously detected with current sequencing procedures (Van Deusen et al. 2025), it remains unknown which functions GFAP serves in iPSCs. GFAP, like other intermediate filament proteins, can exist in a soluble and non‐soluble form. It has been shown that when GFAP becomes phosphorylated on its head domain, it disassembles and localizes to the mitotic spindle during cell division (Matsuoka et al. 1992; Sekimata et al. 1996). Since iPSCs are highly actively dividing cells, GFAP could interact with cell division processes of iPSCs. Moreover, we observed phosphorylated GFAP (Ser13) in AxD iPSCs, where it localized mainly to the nucleus. Interestingly, GFAP has also been shown to be hyperphosphorylated on Ser13 of its head domain in AxD patients, as well as in iPSC‐derived AxD models (Battaglia et al. 2019). Even though mutant GFAP (Viedma‐Poyatos et al. 2022), as well as the absence of GFAP and Vimentin (de Pablo et al. 2013), have been shown to increase susceptibility to oxidative stress, it remains to be seen whether soluble mutant GFAP can affect cell cycle states. In addition, it remains elusive how exactly mutant GFAP causes altered lineage commitment in organoid systems. It could act simply through an increase in cellular stress, or it could disrupt the cytoskeleton in such a way that it can no longer provide the homeostatic regulation of integrating mechanical signals with downstream signaling pathways that regulate differentiation. Mechanosensitive lineage commitment of stem cells has been investigated (Baek, Lopez, et al. 2022; Baek, Kumar, et al. 2022; Keung et al. 2011; Rammensee et al. 2017), but not in the context of GFAP.

Finally, our data show that presumably minor adaptations in embryoid body formation can have strong effects on organoid identity. Indeed, it has been reported that tissue geometry drives deterministic patterning of organoids and that early geometrical confinement of embryoid bodies has an effect on neural lineage specification (Gjorevski et al. 2022; Sen et al. 2021). This further highlights the importance of careful considerations regarding model choice in the context of disease modeling.

To conclude, we show that GFAP is expressed early during embryoid body‐ and organoid formation and that mutations in *GFAP* have a strong effect on lineage specification.

## Author Contributions

W.D., Z.M., and P.A. conducted experiments and analyzed the data. W.D. and Z.M. prepared the figures. W.D., Z.M., and E.M.H. conceptualized and wrote the paper. R.A.B. and L.L. generated the iPSC lines from the R239C patient. Y.S. supervised the generation of the R239C‐AxD‐2 line by L.L. and provided input on the manuscript. D.P.‐S., H.A., N.G.‐I., and J.C. provided valuable input. R.J.P., L.V., M.K., M.P., and E.M.H. supervised the project.

## Conflicts of Interest

The authors declare no conflicts of interest.

## Supporting information


**Figure S1.** Sanger sequencing output of AxD lines (A) Sanger sequencing confirmed the presence of the R239C mutation in both AxD patient‐derived iPSC lines, as well as the correcting of the mutation in the isogenic control line, R239R‐Ctrl, derived from R239C‐AxD‐1.

**Figure S2.** GFAP KO in R239C‐AxD‐2 line shows a rescuing effect (A) Sanger sequencing output revealing a 25‐ bp deletion in exon 1 of *GFAP* in the R239C‐GFAP‐KO iPSC line. (B) Brightfield microscopy images showing 9‐day‐old R239C‐AxD‐2 and R239C‐GFAP‐KO unguided neural organoids. Arrows indicate different morphology of organoid edges. (C) Immunofluorescent microscopy images showing PAX6 and SOX2 expression in 9‐day‐old R239C‐GFAP‐KO unguided neural organoids. (D) Quantification of SOX2 and PAX6 immunofluorescent signal in 9‐day‐old UNOs relative to the number of cells as measured by Hoechst signal. Each datapoint represents the relative signal of one image. One‐way ANOVA with Tukey’s multiple comparisons test: ****p < 0.0001. (E) Western blot for GFAP on 9‐day‐old unguided neural organoids showing the presence of GFAP in R239R‐Ctrl, R239C‐AxD‐1, R239C‐AxD‐2, R88C‐AxD, R416W‐AxD organoids and the lack thereof in R239C‐GFAP‐KO organoids. (F) Total protein stain corresponding to the western blot shown in (D). Size bars = 100 μm. ImageJ processing of western blots is illustrated in Figure S9.

**Figure S3.** Altered expression of key developmental markers in AxD small EB unguided neural organoids and cortical organoids. (A, B) Normalized expression of *PAX6* and *SOX2* in unguided neural organoids (A) at day 9, generated from small (= 4,500 cells) embryoid bodies, and cortical organoids (B), as measured by RT‐qPCR. Expression in all plots is relative to housekeeping genes *GAPDH, ACTB, SDHA, TBP, RPII* and *18S*. Unpaired t‐test: *p < 0.05, **p < 0.01, ***p < 0.001, ****p < 0.0001. Each datapoint represents one batch of organoids.

**Figure S4.** GO terms plots for common DEGs between AxD and Ctrl for the confined vs direct seeding comparison. (A) Up‐(left) and downregulated (right) GO terms for confined (Aggrewell800) compared to direct‐seeding neural organoids (9‐day‐old) for the common DEGs between R239C‐AxD‐1 and R239C‐Ctrl. *p*_adj_ < 0.1.

**Figure S5.** Western blot showing the presence of GFAP in multiple AxD patient‐derived iPSC lines. (A) Western blot showing a band for GFAP (DAKO antibody) at around 55 kDa for multiple iPSC lines, indicated by the arrow. (B) Western blot showing a band for GFAP (SIGMA antibody) at around 50 kDa for multiple iPSC lines, indicated by the arrow. Arrowhead indicates slightly higher band for 100‐day‐old healthy control unguided neural organoids. (C) Total protein stain for the blot that was used in (A) and (B). ImageJ processing of western blots is illustrated in Figures S10 and S11.

**Figure S6.** Sanger sequencing output for potential off‐targets relating to the CRISPR/Cas9 generated AxD‐GFAP‐KO line. Sanger output for AxD‐R239C‐AxD‐2 and AxD‐GFAP‐KO for potential off‐targets *HLA‐F*, *RBFOX1*, *NFIC* and *FABP6*. Red arrows indicate intronic heterozygous mutations in the *FABP6* gene of the AxD‐GFAP‐KO line.

**Figure S7.** Dual SMAD inhibition (DSI) rescues PAX6 expression in neural organoids derived from multiple AxD iPSC lines. (A) Quantification of immunofluorescent signal of PAX6 in 9‐day‐old UNOs. (B) Quantification of immunofluorescent signal of PAX6 in 9‐day‐old organoids, comparing the regular UNO protocol with the dual SMAD inhibition (DSI) protocol. (C) Quantification of immunofluorescent signal of PAX6 in 9‐day‐old organoids made with the DSI protocol. Each datapoint represents one image of one organoid. Area is measured in μm^2^. One‐way ANOVA with Tukey’s correction for multiple testing: ns = not significant, *p < 0.05, ****p < 0.0001.

**Figure S8.** GFAP immunocytochemistry in iPSCs. (A) Immunocytochemistry for GFAP using the GFAP DAKO antibody, showing GFAP signal in healthy control iPSCs, and the lack thereof in a secondary‐only negative control. Size bars = 100 μm. (B) Confocal (maximum intensity projection) microscopy images showing pSer13 GFAP signal in R239R‐Ctrl and R239C‐AxD‐1 iPSCs and the lack thereof in a secondary antibody only (negative control) image. Size bars = 30 μm.

**Figure S9.** Western blot processing corresponding to Figure S2. (A) Western blot processing steps for Figure S2E. (B) Western blot processing steps for Figure S2F. For both blots, images were analyzed in ImageJ software and LUT was inverted to visualize the blot background as white and the blotted protein as black, as follows. Image ➔ color ➔ gray. Image ➔ color ➔ invert LUT, yielding the final images shown in Figure S2E,F.

**Figure S10.** Western blot processing corresponding to Figure S5A,C. (A) Western blot processing steps for Figure S5A using ImageJ software. For the blot belonging to Figure S5A, the red channel showed the protein of interest (GFAP) and not the ladder, whereas the green channel showed the ladder, but not the protein of interest. Therefore, for Figure 5A, we proceeded as follows. Image ➔ color ➔ split ➔ merge red/green channel ➔ LUT ➔ gray for both channels ➔ image ➔ type ➔ RGB color ➔ image ➔ type ➔ 32 bit ➔ invert LUT. (B) Western blot processing steps for Figure 5C. The western blot was analyzed in ImageJ software and LUT was inverted to visualize the blot background as white and the blotted protein as black, as follows. Image ➔ color ➔ gray. Image ➔ color ➔ invert LUT.

**Figure S11.** Western blot processing corresponding to Figure S5B,D. (A) Western blot processing steps for Figure S2E. (B) Western blot processing steps for Figure S2F. For both blots, images were analyzed in ImageJ software and LUT was inverted to visualize the blot background as white and the blotted protein as black, as follows. Image ➔ color ➔ gray. Image ➔ color ➔ invert LUT, yielding the final images shown in Figure S5B,D.

**Table S1.** List of qPCR primers.

**Table S2.** Primers used for PCR and Sanger sequencing.

**Table S3.** PCR primers used to test for off‐target effects.

**Table S4.** List of antibodies.

**Data S1.** Supporting Information.

## Data Availability

The RNA sequencing data have been deposited at NCBI's Gene Expression Omnibus (Edgar et al. 2002) under accession number GSE267992. The code used for preprocessing and analysis of the transcriptomic data is available at the GitHub repository: https://github.com/LabGenExp/hiPSC‐derived_early_AxD_organoids.
