# Supplementary figures and images for: Mutations in GFAP Alter Early Lineage Commitment of Organoids
Source: Glia. 2025 Jul 30;73(11):2167–88. doi: 10.1002/glia.70049 (PMC12436998; doi:10.1002/glia.70049)

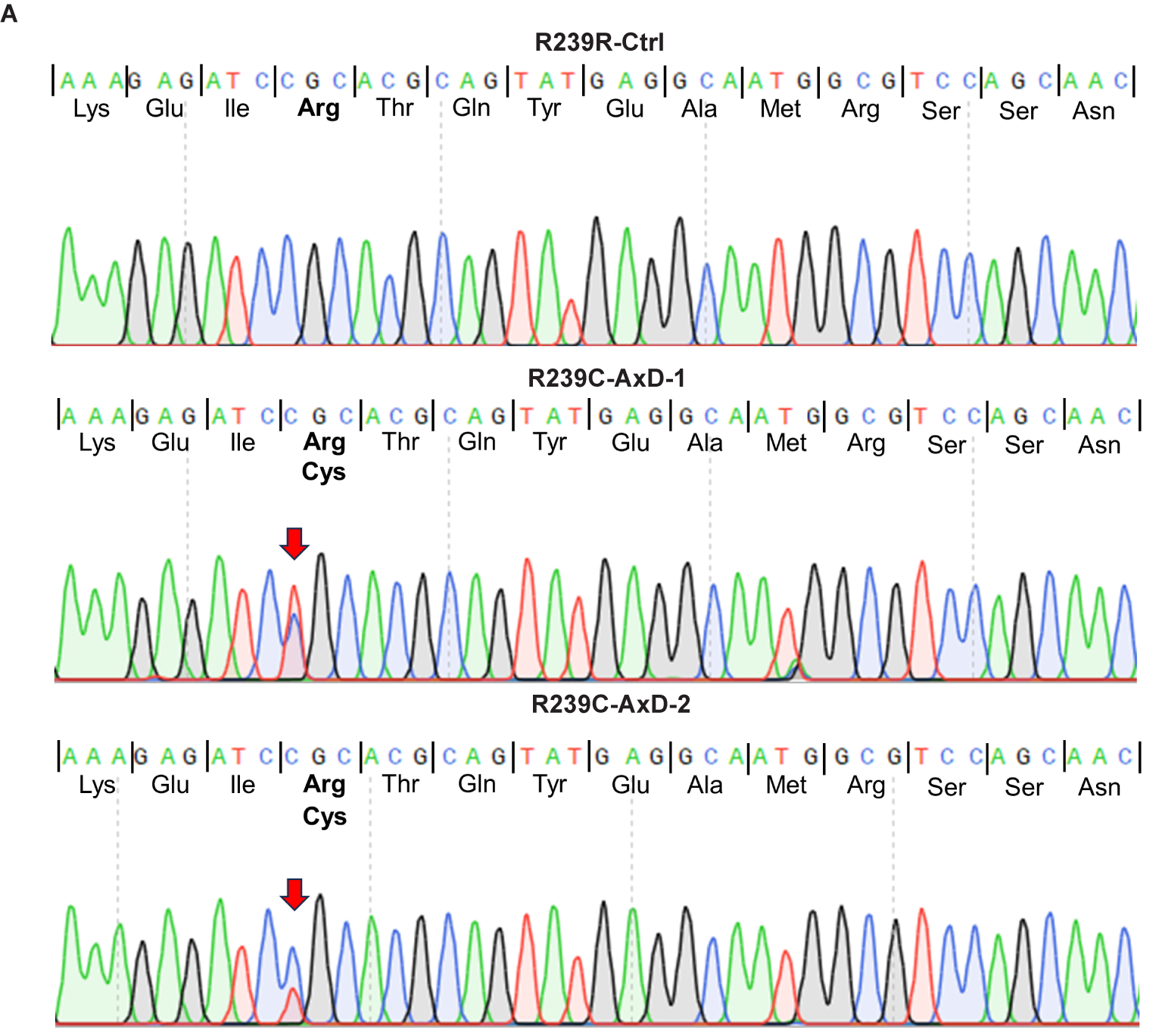

Supplement: Supplementary file 1 — Figure S1. Sanger sequencing output of AxD lines (A) Sanger sequencing confirmed the presence of the R239C mutation in both AxD patient‐derived iPSC lines, as well as the correcting of the mutation in the isogenic control line, R239R‐Ctrl, derived from R239C‐AxD‐1. [file GLIA-73-2167-s003.tif]

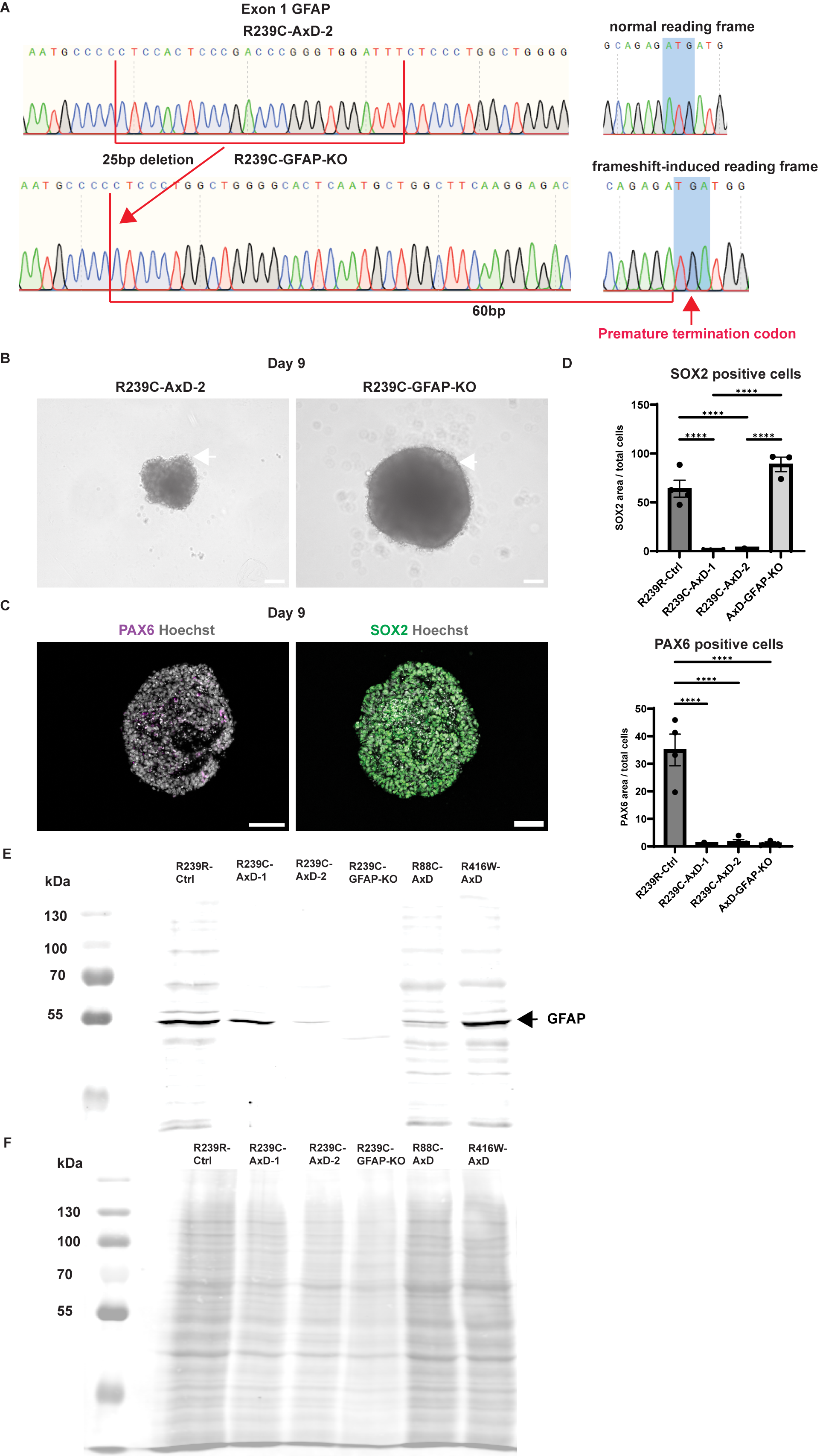

Supplement: Supplementary file 2 — Figure S2. GFAP KO in R239C‐AxD‐2 line shows a rescuing effect (A) Sanger sequencing output revealing a 25‐ bp deletion in exon 1 of GFAP in the R239C‐GFAP‐KO iPSC line. (B) Brightfield microscopy images showing 9‐day‐old R239C‐AxD‐2 and R239C‐GFAP‐KO unguided neural organoids. Arrows indicate different morphology of organoid edges. (C) Immunofluorescent microscopy images showing PAX6 and SOX2 expression in 9‐day‐old R239C‐GFAP‐KO unguided neural organoids. (D) Quantification of SOX2 and PAX6 immunofluorescent signal in 9‐day‐old UNOs relative to the number of cells as measured by Hoechst signal. Each datapoint represents the relative signal of one image. One‐way ANOVA with Tukey’s multiple comparisons test: ****p < 0.0001. (E) Western blot for GFAP on 9‐day‐old unguided neural organoids showing the presence of GFAP in R239R‐Ctrl, R239C‐AxD‐1, R239C‐AxD‐2, R88C‐AxD, R416W‐AxD organoids and the lack thereof in R239C‐GFAP‐KO organoids. (F) Total protein stain corresponding to the western blot shown in (D). Size bars = 100 μm. ImageJ processing of western blots is illustrated in Figure S9. [file GLIA-73-2167-s016.tif]

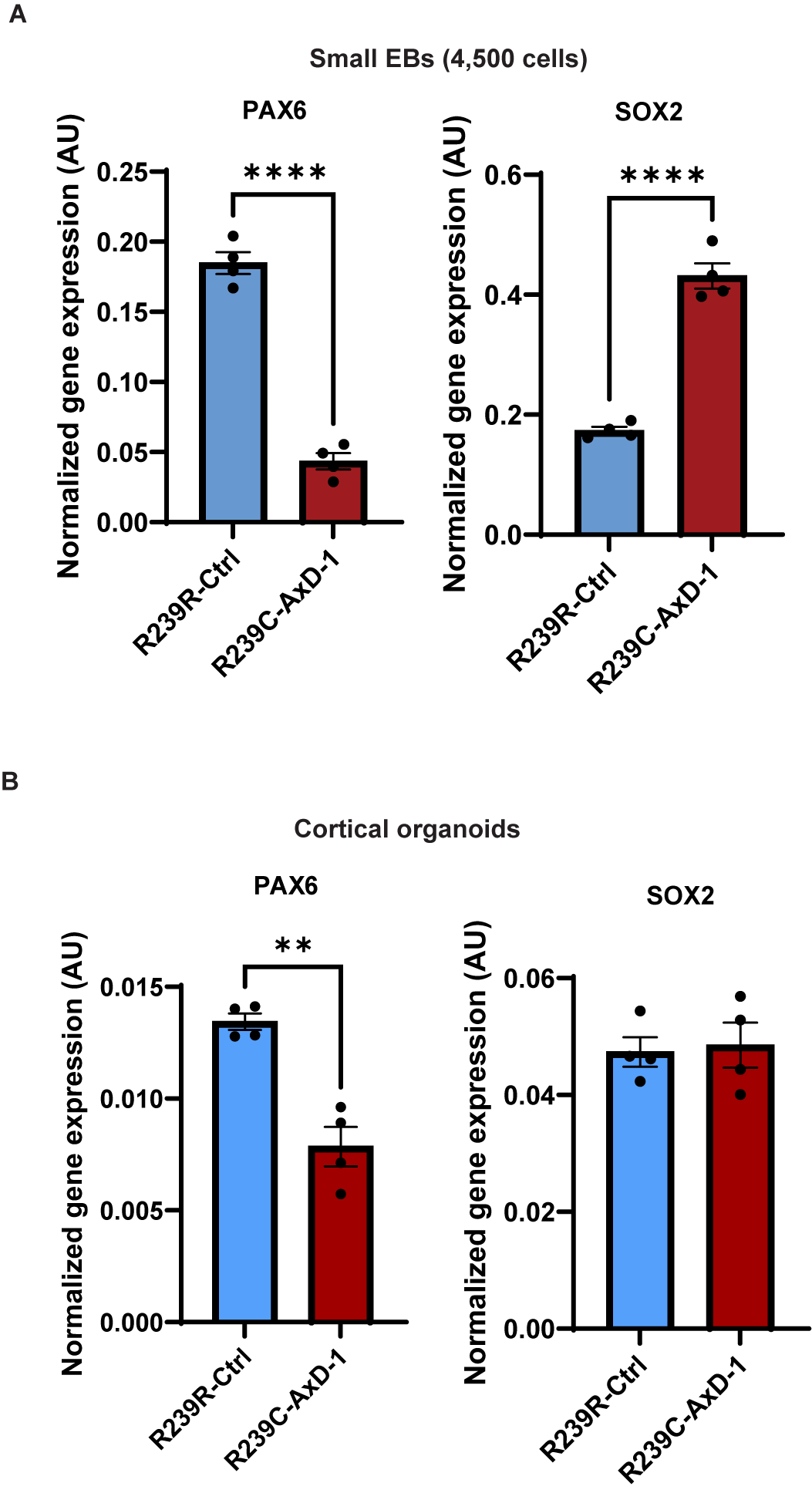

Supplement: Supplementary file 3 — Figure S3. Altered expression of key developmental markers in AxD small EB unguided neural organoids and cortical organoids. (A, B) Normalized expression of PAX6 and SOX2 in unguided neural organoids (A) at day 9, generated from small (= 4,500 cells) embryoid bodies, and cortical organoids (B), as measured by RT‐qPCR. Expression in all plots is relative to housekeeping genes GAPDH, ACTB, SDHA, TBP, RPII and 18S. Unpaired t‐test: *p < 0.05, **p < 0.01, ***p < 0.001, ****p < 0.0001. Each datapoint represents one batch of organoids. [file GLIA-73-2167-s015.tif]

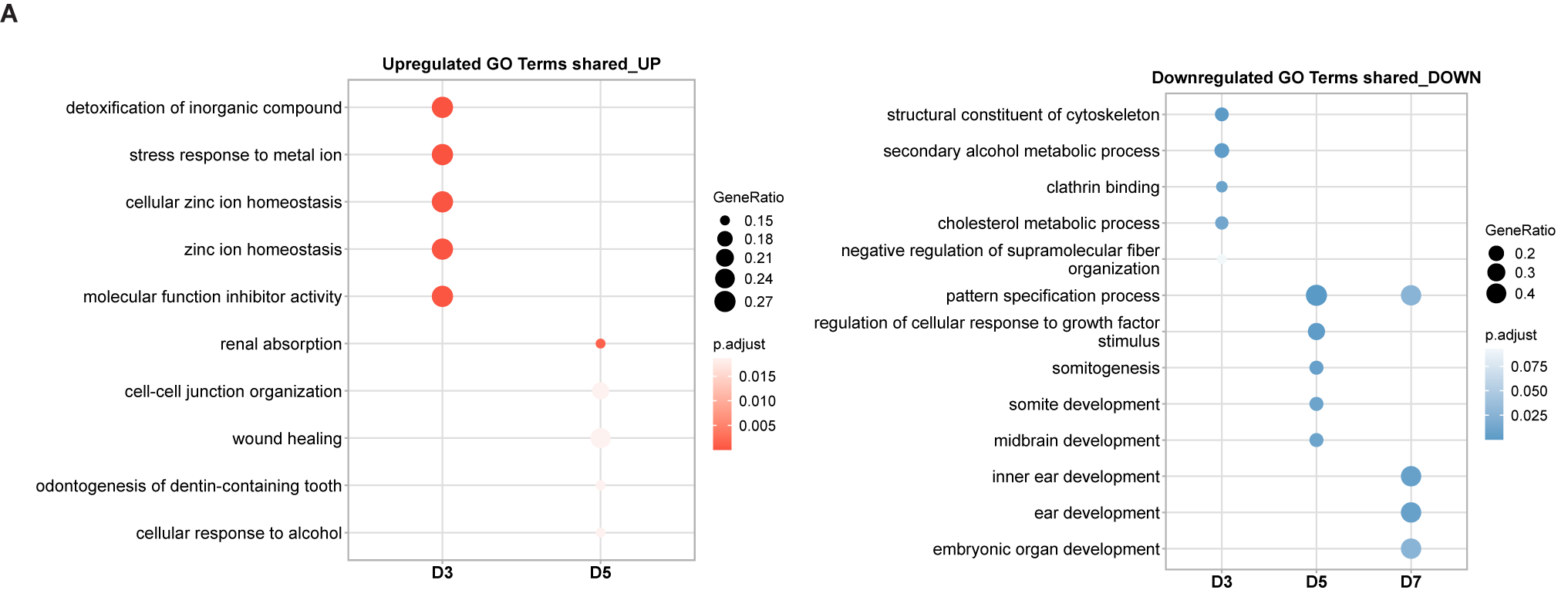

Supplement: Supplementary file 4 — Figure S4. GO terms plots for common DEGs between AxD and Ctrl for the confined vs direct seeding comparison. (A) Up‐(left) and downregulated (right) GO terms for confined (Aggrewell800) compared to direct‐seeding neural organoids (9‐day‐old) for the common DEGs between R239C‐AxD‐1 and R239C‐Ctrl. p adj < 0.1. [file GLIA-73-2167-s013.tif]

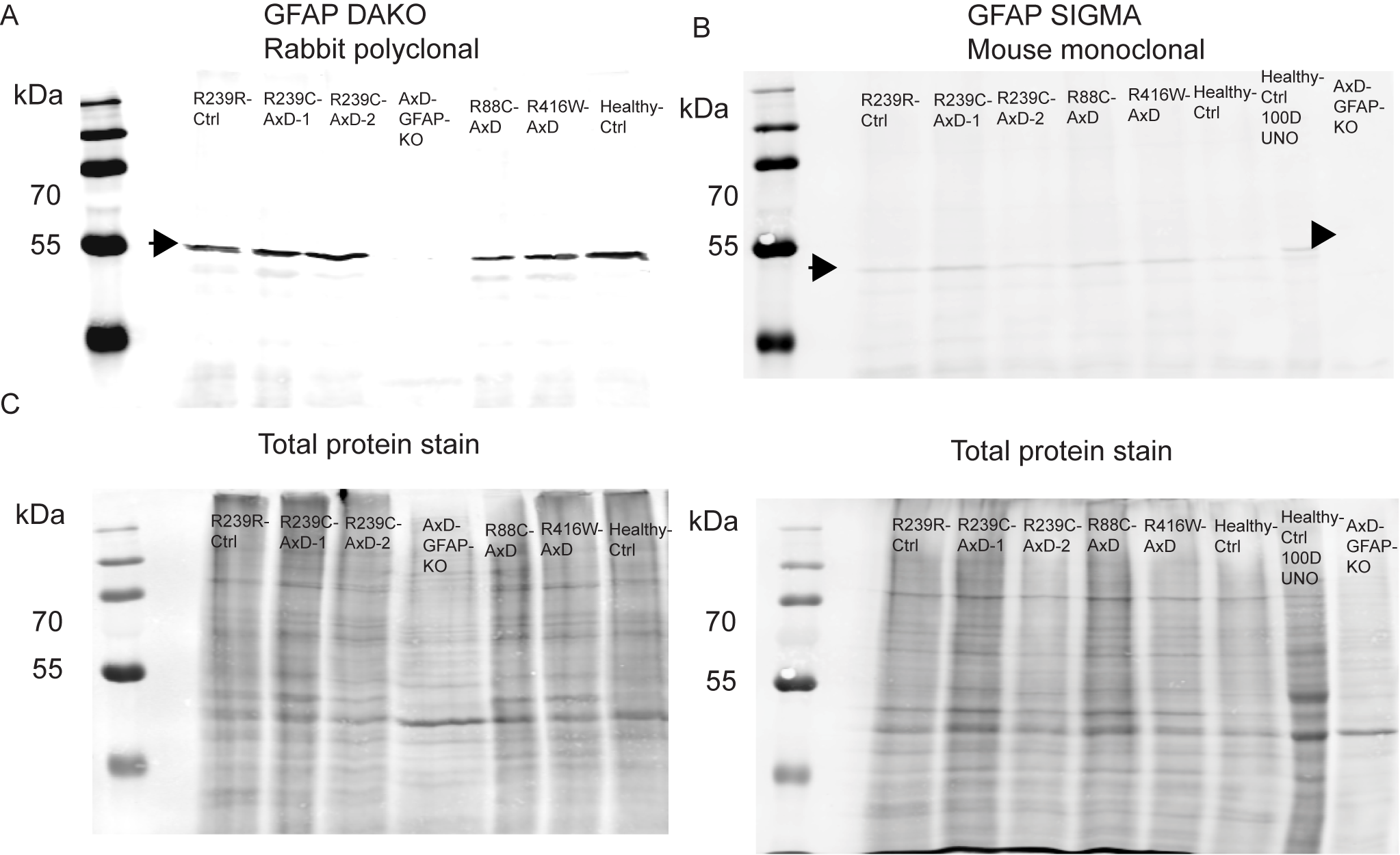

Supplement: Supplementary file 5 — Figure S5. Western blot showing the presence of GFAP in multiple AxD patient‐derived iPSC lines. (A) Western blot showing a band for GFAP (DAKO antibody) at around 55 kDa for multiple iPSC lines, indicated by the arrow. (B) Western blot showing a band for GFAP (SIGMA antibody) at around 50 kDa for multiple iPSC lines, indicated by the arrow. Arrowhead indicates slightly higher band for 100‐day‐old healthy control unguided neural organoids. (C) Total protein stain for the blot that was used in (A) and (B). ImageJ processing of western blots is illustrated in Figures S10 and S11. [file GLIA-73-2167-s009.tif]

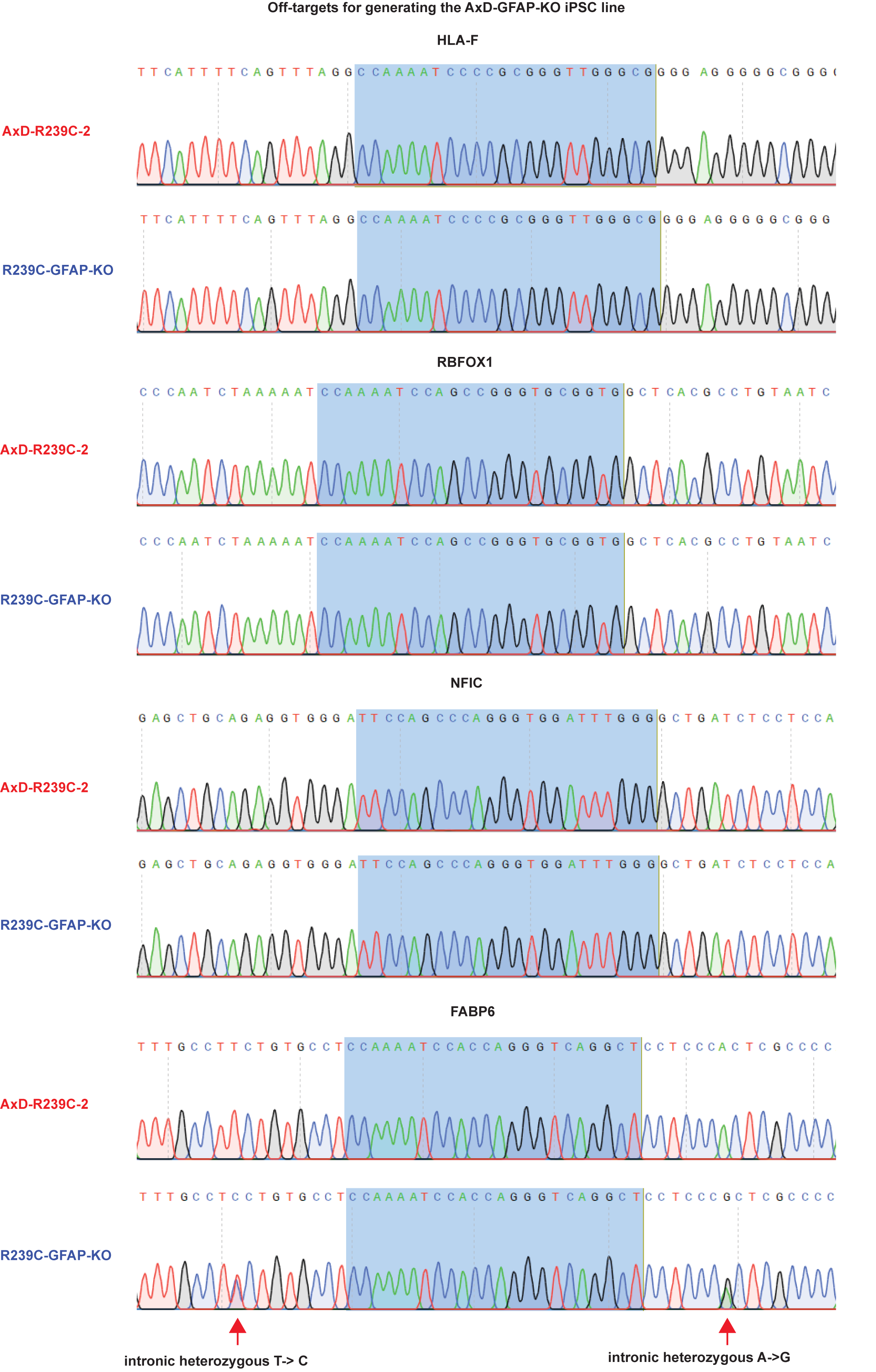

Supplement: Supplementary file 6 — Figure S6. Sanger sequencing output for potential off‐targets relating to the CRISPR/Cas9 generated AxD‐GFAP‐KO line. Sanger output for AxD‐R239C‐AxD‐2 and AxD‐GFAP‐KO for potential off‐targets HLA‐F, RBFOX1, NFIC and FABP6. Red arrows indicate intronic heterozygous mutations in the FABP6 gene of the AxD‐GFAP‐KO line. [file GLIA-73-2167-s008.tif]

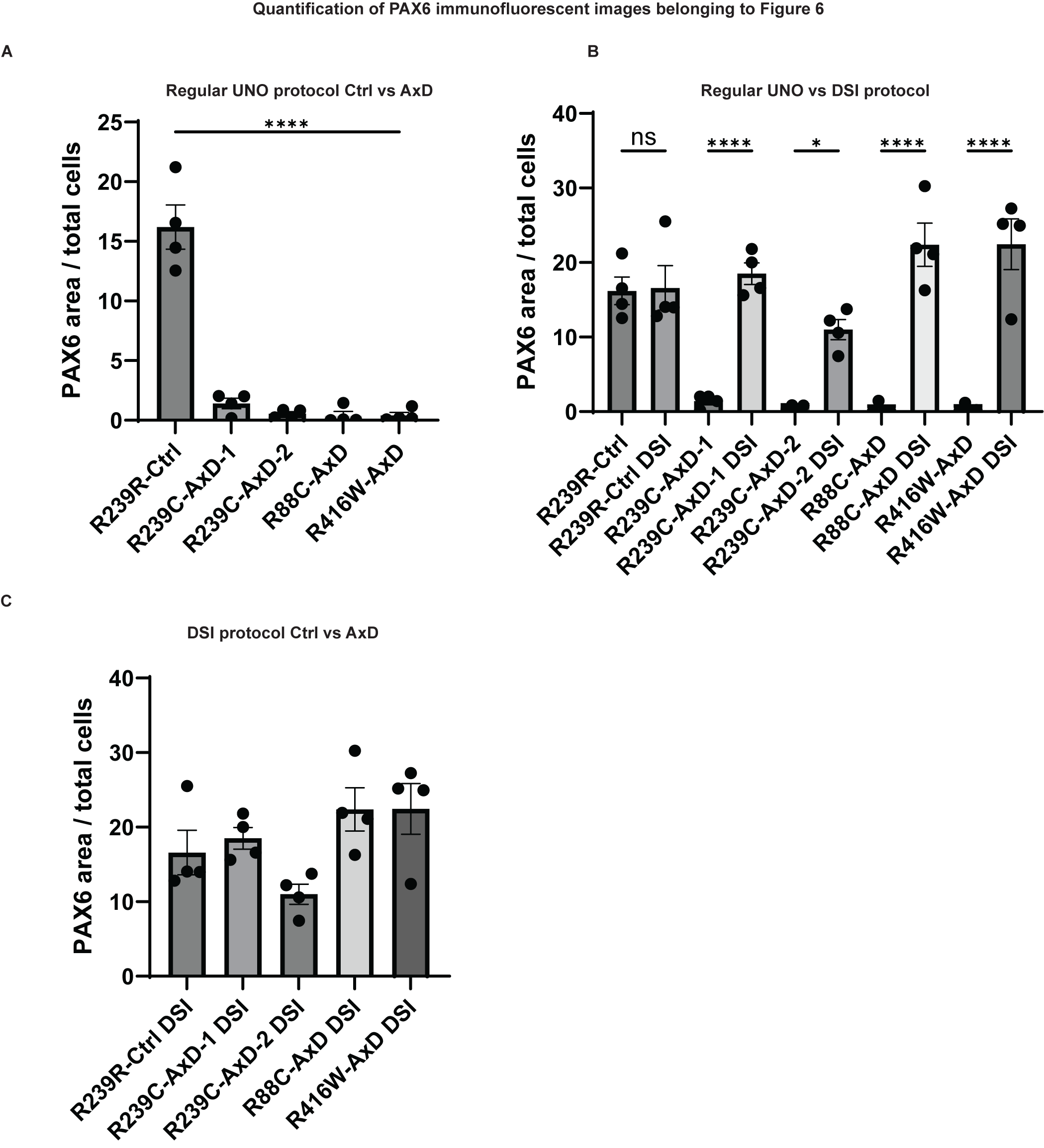

Supplement: Supplementary file 7 — Figure S7. Dual SMAD inhibition (DSI) rescues PAX6 expression in neural organoids derived from multiple AxD iPSC lines. (A) Quantification of immunofluorescent signal of PAX6 in 9‐day‐old UNOs. (B) Quantification of immunofluorescent signal of PAX6 in 9‐day‐old organoids, comparing the regular UNO protocol with the dual SMAD inhibition (DSI) protocol. (C) Quantification of immunofluorescent signal of PAX6 in 9‐day‐old organoids made with the DSI protocol. Each datapoint represents one image of one organoid. Area is measured in μm2. One‐way ANOVA with Tukey’s correction for multiple testing: ns = not significant, *p < 0.05, ****p < 0.0001. [file GLIA-73-2167-s014.tif]

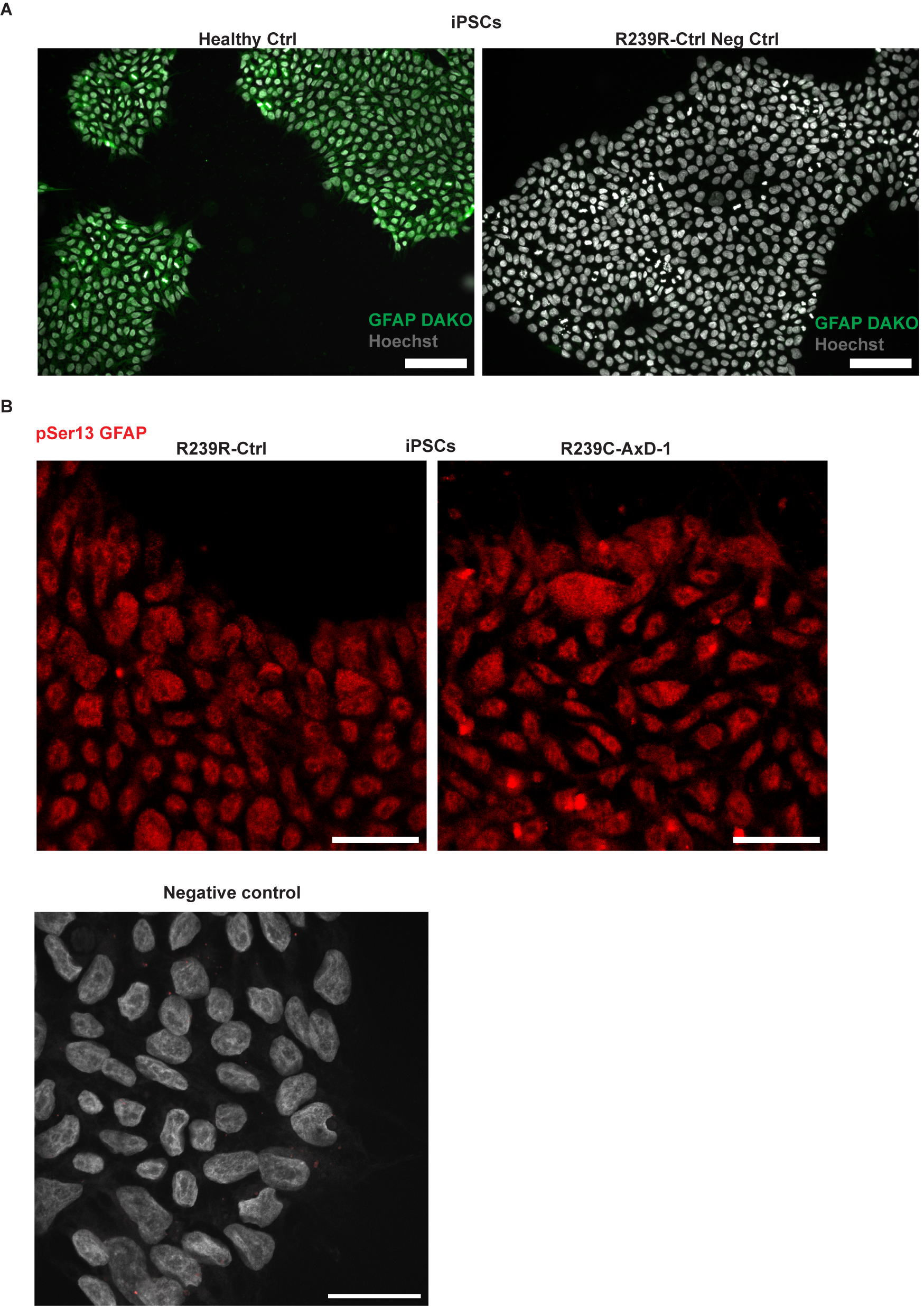

Supplement: Supplementary file 8 — Figure S8. GFAP immunocytochemistry in iPSCs. (A) Immunocytochemistry for GFAP using the GFAP DAKO antibody, showing GFAP signal in healthy control iPSCs, and the lack thereof in a secondary‐only negative control. Size bars = 100 μm. (B) Confocal (maximum intensity projection) microscopy images showing pSer13 GFAP signal in R239R‐Ctrl and R239C‐AxD‐1 iPSCs and the lack thereof in a secondary antibody only (negative control) image. Size bars = 30 μm. [file GLIA-73-2167-s001.tif]

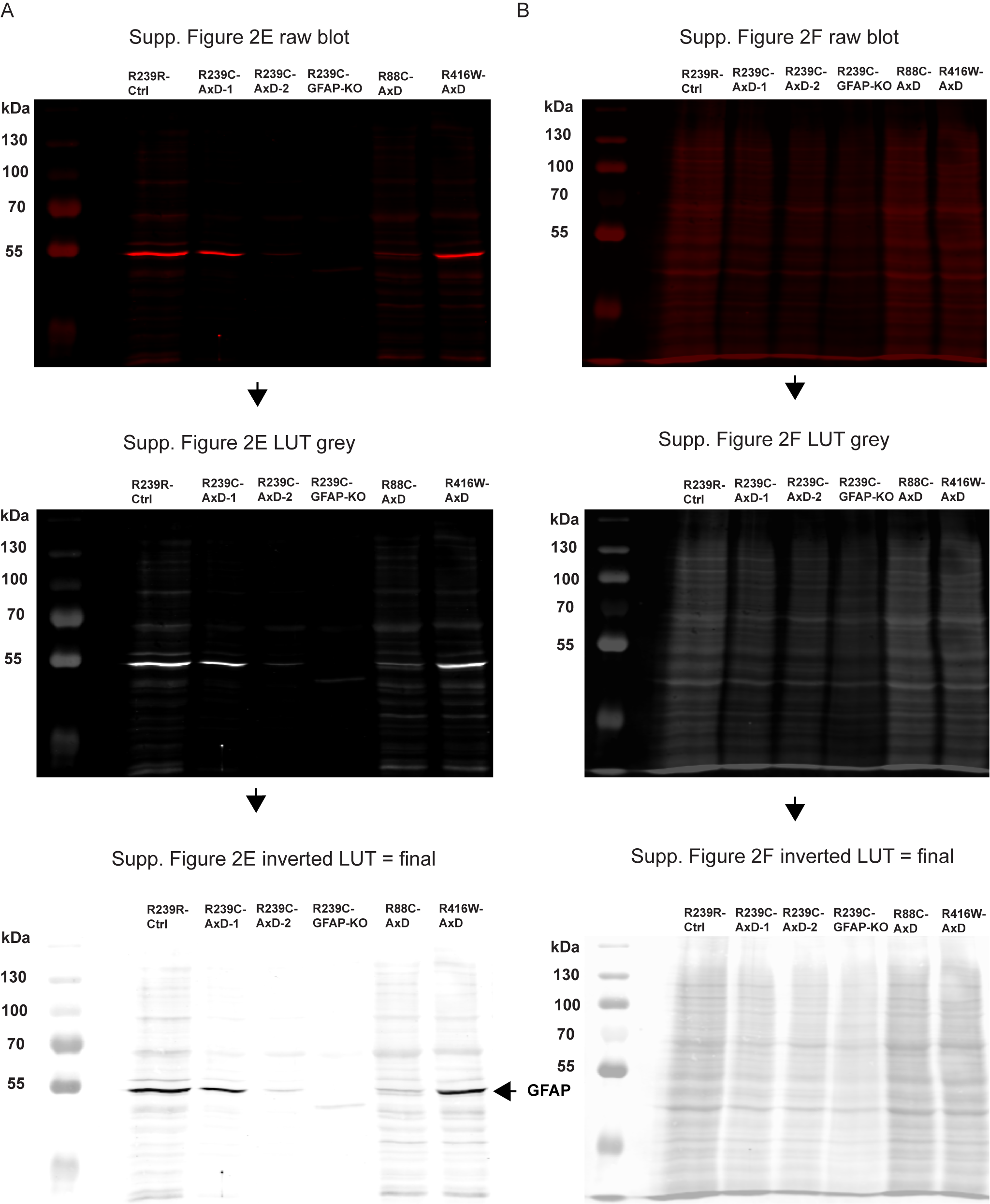

Supplement: Supplementary file 9 — Figure S9. Western blot processing corresponding to Figure S2. (A) Western blot processing steps for Figure S2E. (B) Western blot processing steps for Figure S2F. For both blots, images were analyzed in ImageJ software and LUT was inverted to visualize the blot background as white and the blotted protein as black, as follows. Image ➔ color ➔ gray. Image ➔ color ➔ invert LUT, yielding the final images shown in Figure S2E,F. [file GLIA-73-2167-s004.tif]

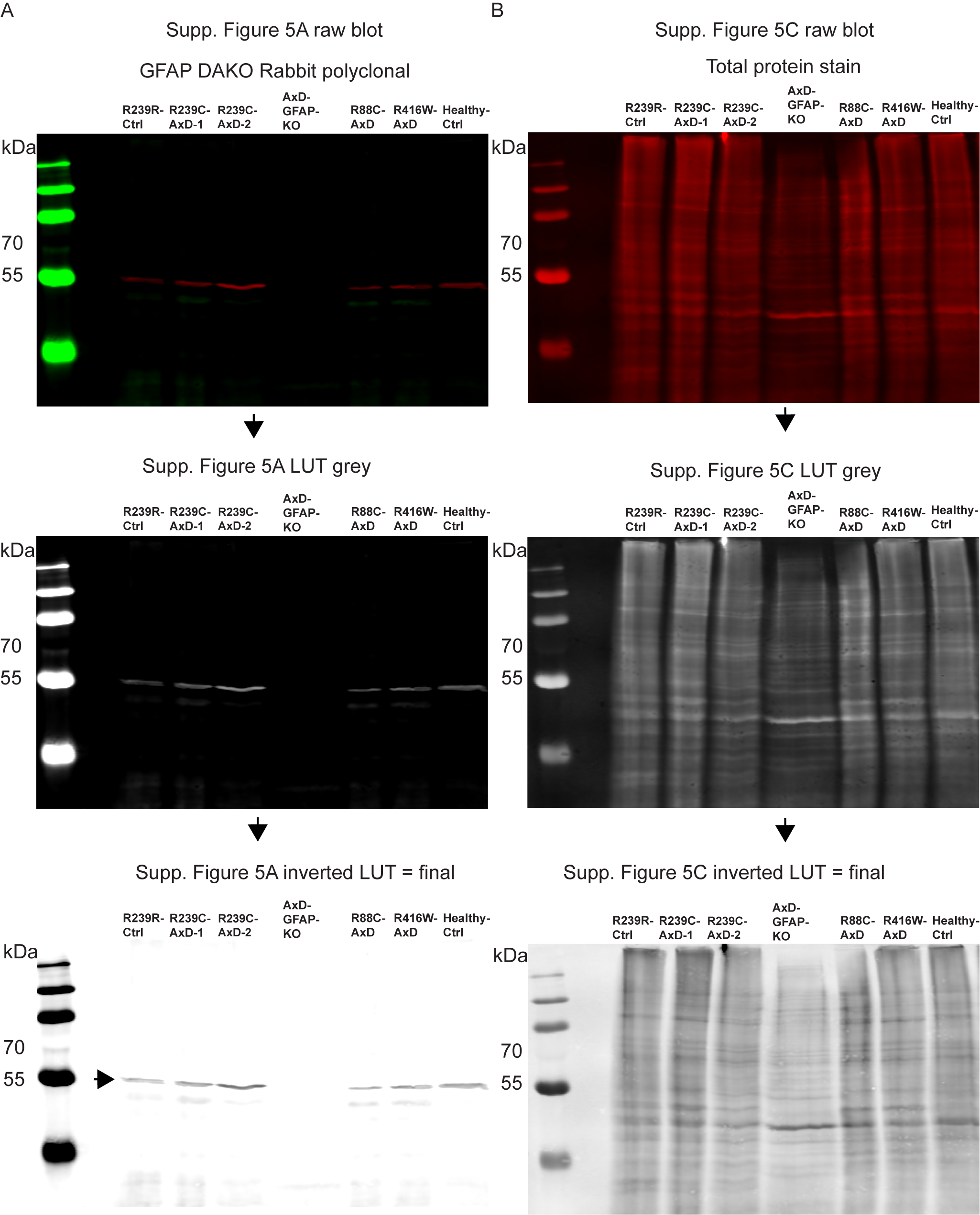

Supplement: Supplementary file 10 — Figure S10. Western blot processing corresponding to Figure S5A,C. (A) Western blot processing steps for Figure S5A using ImageJ software. For the blot belonging to Figure S5A, the red channel showed the protein of interest (GFAP) and not the ladder, whereas the green channel showed the ladder, but not the protein of interest. Therefore, for Figure 5A, we proceeded as follows. Image ➔ color ➔ split ➔ merge red/green channel ➔ LUT ➔ gray for both channels ➔ image ➔ type ➔ RGB color ➔ image ➔ type ➔ 32 bit ➔ invert LUT. (B) Western blot processing steps for Figure 5C. The western blot was analyzed in ImageJ software and LUT was inverted to visualize the blot background as white and the blotted protein as black, as follows. Image ➔ color ➔ gray. Image ➔ color ➔ invert LUT. [file GLIA-73-2167-s012.tif]

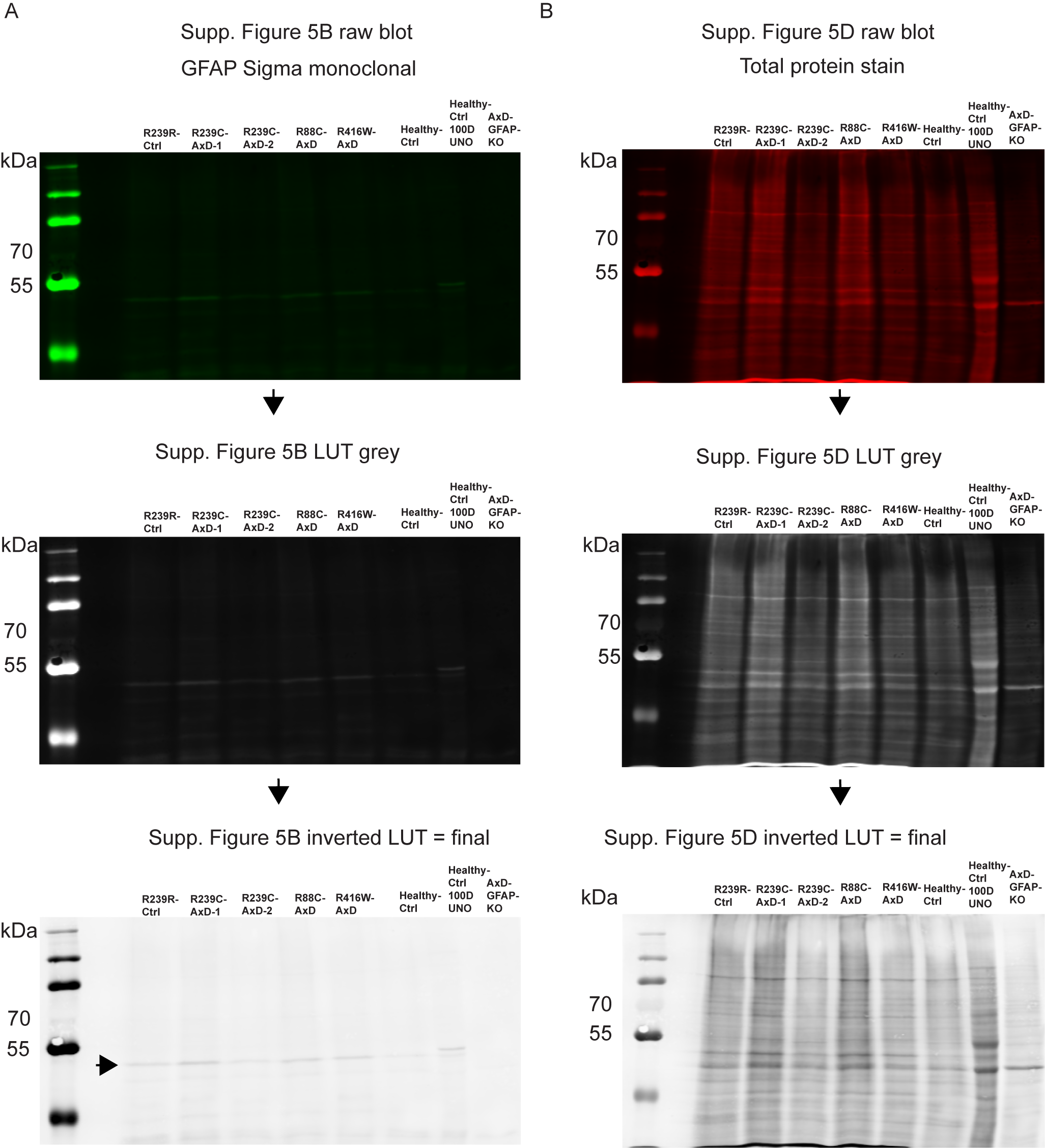

Supplement: Supplementary file 11 — Figure S11. Western blot processing corresponding to Figure S5B,D. (A) Western blot processing steps for Figure S2E. (B) Western blot processing steps for Figure S2F. For both blots, images were analyzed in ImageJ software and LUT was inverted to visualize the blot background as white and the blotted protein as black, as follows. Image ➔ color ➔ gray. Image ➔ color ➔ invert LUT, yielding the final images shown in Figure S5B,D. [file GLIA-73-2167-s005.tif]
