# Supplementary material for: Mutations in GFAP Alter Early Lineage Commitment of Organoids
Source: Glia. 2025 Jul 30;73(11):2167–88. doi: 10.1002/glia.70049 (PMC12436998; doi:10.1002/glia.70049)
Supplement: Supplementary file 12 — Table S1. List of qPCR primers. [file GLIA-73-2167-s011.docx]

**Supplementary table 1**. *List of qPCR primers*

| **Gene** | **Forward (5’🡪3’)** | **Reverse (5’🡪3’)** |
| --- | --- | --- |
| GAPDH | TGCACCACCAACTGCTTAGC | GGCATGGACTGTGGTCATGA |
| B-actin | GTGGACATCCGCAAAGACCT | TCTGCATCCTGTCGGCAAT |
| TBP | CCACAGCTCTTCCACTCACA | GCGGTACAATCCCAGAACTC |
| SDHA | GAAGCCCTTTGAGGAGCACT | GTTTTGTCGATCACGGGTCT |
| RPII | GCACCACGTCCAATGACAT | GTGCGGCTGCTTCCATAA |
| 18S | TAGTCGCCGTGCCTACCAT | CCTGCTGCCTTCCTTGGA |
| PAX6 | TGGGCAGGTATTACGAGACTG | ACTCCCGCTTATACTGGGCTA |
| SOX2 | ATGCACCGCTACGACGTGAG | GCGAGTAGGACATGCTGTAGG |
| BMP4 | ATGATTCCTGGTAACCGAATGC | CCCCGTCTCAGGTATCAAACT |
| TNNT2 | TTCGACCTGCAGGAGAAGTT | GCGGGTCTTGGAGACTTTCT |
