# Supplementary material for: Mutations in GFAP Alter Early Lineage Commitment of Organoids
Source: Glia. 2025 Jul 30;73(11):2167–88. doi: 10.1002/glia.70049 (PMC12436998; doi:10.1002/glia.70049)
Supplement: Supplementary file 13 — Table S2. Primers used for PCR and Sanger sequencing. [file GLIA-73-2167-s007.docx]

**Supplementary table 2.** *Primers used for PCR and Sanger sequencing*

| **Gene** | **5’🡪3’** | **Annealing temp. (°C)** |
| --- | --- | --- |
| GFAP intron 3 | CTGGTACCGCTTCTCTCACC | 58 |
| GFAP intron 4 | CAGCTTCTTCCACCCTCC | 58 |
| GFAP exon 1-2 | AGGCAGAAGCTCCAGGATGA | 58 |
| GFAP exon 5 | GGTCGCAGGTCAAGGACTG | 58 |
