# Supplementary material for: Mutations in GFAP Alter Early Lineage Commitment of Organoids
Source: Glia. 2025 Jul 30;73(11):2167–88. doi: 10.1002/glia.70049 (PMC12436998; doi:10.1002/glia.70049)
Supplement: Supplementary file 14 — Table S3. PCR primers used to test for off‐target effects. [file GLIA-73-2167-s006.docx]

**Supplementary Table 3.** PCR primers used to test for off-target effects

| **Gene** | **Target sequence** | **Primer** | **5´ 🡪 3´** | **Annealing temp. (°C)** |
| --- | --- | --- | --- | --- |
| FABP6 | agCCtGACCCtGGTGGATTT-TGG | Forward | ATGATTCAGGCCAGAAGGGG | 55°C |
|  |  | Reverse | GCTTTGGAGCCATGACAAGT |  |
| NFIC | tTCCaGcCCaGGGTGGATTT-GGG | Forward | CCAGTTCCTTTGGGTGACCT | 55°C |
|  |  | Reverse | TGCAGTCAACGCTCCCATTT |  |
| RBFOX1 | CaCCgcACCCGGcTGGATTT-TGG | Forward | TTGGTGCCACAAGGAACAGT | 55°C |
|  |  | Reverse | TGACTTCCAGATTGGTGAGATTC |  |
| HLA-F | CgCCCaACCCGcGgGGATTT-TGG | Forward | ACGCACAGACTGACCGAGT | 55°C |
|  |  | Reverse | ACGCACAGACTGACCGAGT |  |
|  |  |  |  |  |

Note. Base pairs that differ between the guide RNA and target sequence of possible off-target genes are marked red and in lower-case. Primers were designed to include the target sequence.
