# Supplementary material for: Mutations in GFAP Alter Early Lineage Commitment of Organoids
Source: Glia. 2025 Jul 30;73(11):2167–88. doi: 10.1002/glia.70049 (PMC12436998; doi:10.1002/glia.70049)
Supplement: Supplementary file 15 — Table S4. List of antibodies. [file GLIA-73-2167-s002.docx]

**Supplementary table 4**. *List of antibodies*

| **Antigen** | **Host** | **Manufacturer** | **Catalog No.** | **Dilution** |
| --- | --- | --- | --- | --- |
| GFAP | Rabbit | Agilent, DAKO | Z0334 | 1:1000 (IHC) / 1:5000 (WB) |
| GFAP | Mouse | Sigma | G3893 | 1:500 (WB) |
| Phospho13-GFAP | Mouse | A generous gift from Dr. Inagaki |  | 1:125 (IHC) |
| PAX6 | Rabbit | ThermoFisher Scientific | 42-6600 | 1:500 |
| SOX2 | Rabbit | Merck Chemicals | AB5603 | 1:1000 |
| Anti-rabbit, Alexa Fluor 488 | Donkey | Jackson Immunoresearch | 711-545-152 | 1:1000 |
| Anti-rabbit Alexa Fluor 568 | Donkey | Abcam | Ab175470 | 1:1000 |
| Anti-mouse Alexa Fluor 568 | Donkey | Life Technologies | A10037 | 1:1000 |

Abbreviations: IHC = immunohistochemistry, WB = western blot
